# Degradable Nanocarriers Capable of Gene Delivery Derived from pH‐Responsive Polyester: RROP Copolymerization Between Cyclic Ketene Acetals

**DOI:** 10.1002/marc.202500722

**Published:** 2025-10-16

**Authors:** Yiyi Deng, Jonas Debbeler, Anja Traeger, Jens Gaitzsch

**Affiliations:** ^1^ Leibniz‐Institut Für Polymerforschung Dresden e.V. Dresden Germany; ^2^ Fakultät Chemie und Lebensmittelchemie Technische Universität Dresden Dresden Germany; ^3^ Université Paris‐Saclay CNRS Institut Galien Paris‐Saclay Orsay France; ^4^ Laboratory of Organic and Macromolecular Chemistry Friedrich Schiller University Jena Jena Germany; ^5^ Jena Center for Soft Matter (JCSM) Friedrich Schiller University Jena Jena Germany

**Keywords:** biodegradable polyesters, cyclic ketene acetals (CKAs), pH‐responsive nanoparticles, gene delivery, radical ring‐opening polymerization (RROP)

## Abstract

The ability to combine biodegradability with responsive units by radical ring‐opening polymerization (RROP) is the ideal setup for a modern drug delivery system. Since the required polymers were recently unlocked by RROP copolymerization between different cyclic ketene acetals (CKAs), we herein prepared a series of polyester copolymers derived from an amine‐bearing CKA 6‐(*iso*‐propyl)‐2‐methylene‐1,3,6‐dioxazocane (*^i^* Pr‐MAC) and 2‐methylene‐1,3,6‐trioxocane (MTC). Functional nanoparticles with tunable sizes and extremely narrow size distributions could be achieved from these copolymers. Comprehensive studies showed that the resulting nanoparticles exhibited tuneable pH‐transition points from 6.3 to 7.1 as the *^i^* Pr‐MAC content increased from 10 to 40 mol%, enabling precise pH‐triggered control release at varying pH levels. Furthermore, this work demonstrates the potential of these pH‐sensitive polyesters as an alternative to traditional polycations, exploring their biocompatibility and gene delivery capability, which suggests a new application prospect for CKA‐derived functional polyesters.

## Graphical abstract

Polyesters with inherent pH‐sensitivity are prepared through radical ring‐opening polymerization between cyclic ketene acetals. These polyesters demonstrate great potential as alternatives to conventional polycations for fabricating hybrid nanoparticles. The obtained nanoparticles exhibit good biocompatibility and are capable of binding and transferring genetic materials into cells.

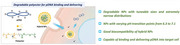

## 1 Introduction

Vinyl‐based functional polymers have been extensively synthesized and studied due to their versatile functional groups and the ease of post‐modification, allowing them to respond to exogenous/endogenous stimuli, such as temperature [1, 2, 3], pH value [3, 4, 5], redox [3, 6, 7], light [3, 8], etc. Among these, pH‐responsive vinyl polymers—and their derived nanocarriers, particularly—attracted considerable interest. Owing to the distinct pH levels in tissues, blood, and intracellular compartments [9, 10, 11], these nanocarriers are ideal as their responsive windows fall within these pH ranges (7.4 to 5). Therefore, designing nanocarriers that selectively respond to pH changes for targeted delivery and controlled release under specific biological conditions is essential. However, the very persistent C─C backbones of polyvinylenes resist degradation in biological environments, resulting in deleterious bioaccumulation and can cause long‐term toxicity [12]. As they are readily biodegradable, aliphatic polyesters remain predominantly used for biomedical applications today, despite limited functional groups and the need for post‐polymerization‐modification [13, 14, 15, 16, 17].

Radical ring‐opening polymerization (RROP) offers a facile way to randomly insert labile groups, such as esters [18, 19, 20] and thioesters [21, 22, 23], into C─C polymer backbone through copolymerization of cyclic monomers, such as cyclic ketene acetals (CKA) and thionolactones with vinylenes [12, 13, 24, 25, 26]. This strategy mitigates the conflict of diverse functionalities and excellent (bio)degradability [27], rendering the obtained copolymers promising candidates to construct nanocarriers through (polymerization‐induced) self‐assembly [23, 28, 29, 30], nanoprecipitation [31, 32], or emulsion polymerization [33, 34, 35]. RROP copolymerization of CKA with vinylenes has been a long focus, but has the significant disadvantage that the great imbalance of reactivity ratios results in unevenly distributed ester units along the polymer chains [24]. RROP homopolymerization of CKAs can produce poly(CKA)s that exhibit complete degradation [36]; however, there is a long‐standing challenge of incorporating efficient functional groups into CKAs, resulting in a major drawback of this approach [37, 38, 39].

This limitation was initially overcome by our group as we reported amine‐bearing CKAs (Alk‐MACs) as a new class of functional CKAs [40]. Following their introduction, the first entirely RROP‐based pH‐responsive polyesters were accessible through homopolymerization of 6‐(*iso*‐propyl)‐2‐methylene‐1,3,6‐dioxazocane (*^i^* Pr‐MAC). In a subsequent work, RROP copolymerization between Alk‐MACs and 2‐methylene‐1,3,6‐trioxocane (MTC) yielded polyester copolymers with varying cloud points ranging from pH 5.7 to 7.2, alongside reversible protonation‐deprotonation behavior and superior hydrolytic degradability [41]. These attractive features position these novel polyesters as promising candidates for fabricating pH‐responsive and degradable nanocarriers optimized for cellular microenvironments with variable pH values.

Furthermore, the poly(Alk‐MAC)s exhibit cationic characteristics within acidic environments. This inherent charge suggests their capability to form complexes with anionic biomolecules, such as plasmid‐DNA (pDNA). Consequently, this highlights their potential utility in gene delivery applications. While poly(β‐amino ester)s (PBAEs) remain a benchmark in degradable polymer‐based gene delivery, their synthesis typically relies on stepwise Michael‐type additions, which limit structural diversity [42, 43, 44]. In contrast, RROP provides a versatile route to pH sensitive biodegradable polyesters, enabling one‐pot incorporation of amine bearing monomers and more flexible tuning of backbone composition and side‐chain chemistry. The transfer of genetic material is accompanied by the need to transfer it across cell membranes into the cytosol and, in the case of pDNA, into the nucleus in order to achieve gene expression. PDNA is an interesting class of therapeutic nucleic acids, enabling the integration of larger gene sequences, e.g., Sleeping Beauty transposons [45]. To achieve an effective route of gene transfer, it is essential to inhibit the degradation of genetic material during its delivery to the target cell and to enable the crossing of the cellular and endosomal membrane [46]. Polymers enable the protection and delivery of genetic material by the formation of nanoparticles (NPs) through electrostatic and/or hydrophobic‐hydrophobic interactions [47, 48]. While gene transfer methods have the potential to treat various diseases and are known to be used as vaccines to prevent diseases [48, 49], innovative materials are required to address current limitations (immune activation, liver clearance, degradability). A central challenge in designing cationic polymers for gene transfer lies in balancing efficient nucleic acid binding with minimal cytotoxicity. By fine‐tuning the ratio of cationic and hydrophobic functionalities, it becomes possible to modulate the pKa of the polymer and achieve a more favorable compromise between biocompatibility and transfection efficiency. While other biodegradable polymers, such as polysaccharides or polypeptides, are often assumed to be biocompatible, this is not automatically the case when cationic groups are included, yet it is essential for their practical use in biological applications [50, 51, 52, 53, 54, 55, 56, 57]. While pH‐responsive vinyl polymers have been reported in this field, the application of purely CKA‐derived functional polyesters in this context has remained untapped, which sparked our research interest.

**FIGURE 1:**
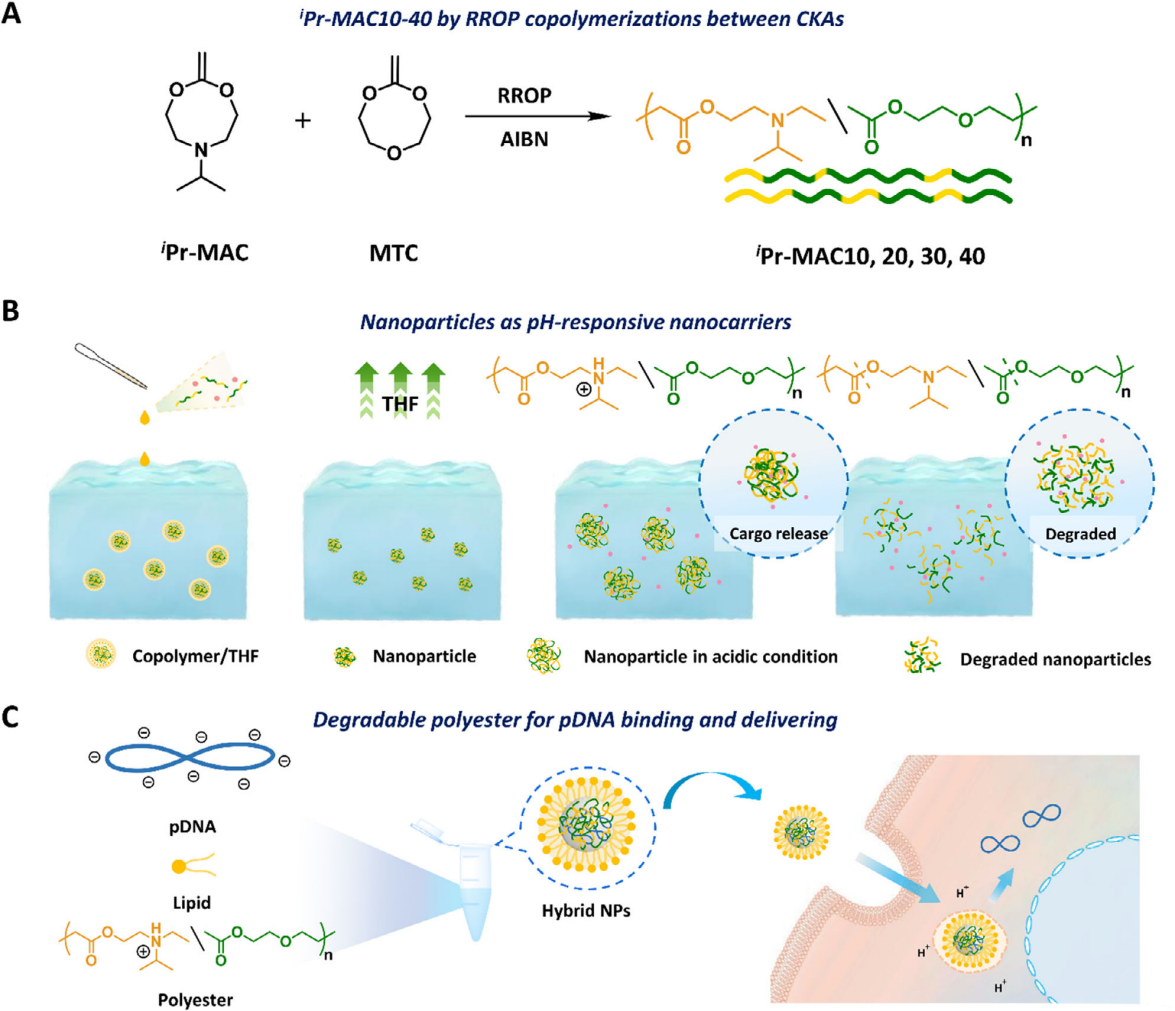
(A) pH‐Responsive and degradable copolyesters with varying *F*(*^i^* Pr‐MAC)/*F*(MTC) yielded through RROP copolymerization within CKAs. Schematic illustrations of (B) preparing pH‐sensitive and degradable NPs by a simple nanoprecipitation method, and cargo release triggered by acidic pH and enzymatic degradation; (C) positively charged polyester copolymers bind with negatively charged pDNA in an acidic environment, and then pH is adjusted to neutral, yielding stable polyester/pDNA complexes. The polyester copolymers act as a degradable template, ensuring pDNA release in an acidic environment, where protonation of the polyester renders the transition from hydrophobic to hydrophilic, facilitating the delivery of the bound pDNA into target cells.

Herein, facile RROP copolymerization between *^i^* Pr‐MAC with MTC is applied to yield polyester copolymers, followed by their nanoprecipitation to yield the desired NPs (Figure 1A). Notably, the molar fraction ratios of *^i^* Pr‐MAC/MTC are well‐modulated. This careful adjustment ensures NPs are capable of responding to varying pH levels and enhances their performance under different biological environments. Comprehensive tests further characterize the resulting NPs, including their sizes, pH‐responsiveness, pH‐triggered release capability, biocompatibility, and hydrolytic degradability (Figure 1B). Finally, the influence of amine content and formulation on nanoparticle formation and delivery to cells, a prerequisite for efficient gene transfer in biomedical contexts, can be demonstrated (Figure 1C). This work presents the initial investigation into the application of novel CKA‐based polyester materials as potential alternatives to traditional cationic vinyl polymers in gene therapy applications utilizing hybrid nanoparticle (hNP) formulations.

## 2 Results and Discussion

The starting CKAs (*^i^* Pr‐MAC and MTC) were individually prepared according to the published carbonate and haloacetal protocol (details in the Supporting Information), respectively. RROP copolymerizations between these two CKAs were performed under thermal initiation (Table S1 and Figure S3). Target molar fractions of *^i^* Pr‐MAC (*F*(*^i^* Pr‐MAC)) varied at intervals of 10 mol%, ranging from 10 to 40 mol%. Despite conducting copolymerization under identical *f*(*^i^* Pr‐MAC)/*f*(MTC) conditions, slight differences in the chemical composition of the final products are the consequence of free radical polymerization. For example, copolymerizations at a *f*(*^i^* Pr‐MAC)/*f*(MTC) of 15/85 (mol/mol%) led to slightly different *F*(*^i^* Pr‐MAC)/*F*(MTC) ratios of 18/82 and 24/76 (mol/mol%), respectively (Tables 1, S1 and Figure S4, entry 4‐5). For clarity, all copolymers with similar chemical compositions are grouped and designated according to their *F*(*^i^* Pr‐MAC) content, termed *^i^* Pr‐MAC10/20/30/40. In the ^1^H and ^13^C NMR spectra of the purified copolymers, all resonances of the poly(MTC) main chain and key resonances of the P(*^i^* Pr‐MAC) units (amine and ester groups) could be clearly assigned (Figures S5 and S6), confirming the successful copolymerization.

**TABLE 1:** Poly(*^i^* Pr‐MAC‐co‐MTC) copolymers with varying chemical compositions and the characterization on molecular weight, dispersity, and the sizes of nanoprecipitated NPs from these copolymers.

	*F*(MAC)/*F*(MTC)^a^	*M*_n_ (kg/mol)^b^	*M*_w_ (kg/mol)^b^	*Ð* ^b^	*D*_i_ (nm)^c^	*D*_n_ (nm)^c^	PDI^c^
*^i^* Pr‐MAC10	11/89	12.3	24.9	2.1	429 ± 76	358 ± 69	0.12 ± 0.09
	9/91	30.0	63.0	2.1	482 ± 20	397 ± 36	0.14 ± 0.01
	13/87	49.0	73.8	1.5	315 ± 56	230 ± 35	0.12 ± 0.03
*^i^* Pr‐MAC20	24/76	4.6	14.5	3.2	658 ± 19	597 ± 29	0.11 ± 0.05
	18/82	15.7	40.4	2.4	314 ± 63	247 ± 41	0.11 ± 0.09
*^i^* Pr‐MAC30	31/69	16.4	37.0	2.3	302 ± 130	263 ± 118	0.08 ± 0.09
	32/68	25.4	46.5	1.8	316 ± 79	233 ± 82	0.16 ± 0.04
*^i^* Pr‐MAC40	42/58	4.5	20.2	4.5	195 ± 15	162 ± 12	0.04 ± 0.02
	44/56	19.5	42.0	2.2	243 ± 78	173 ± 21	0.08 ± 0.0
	39/61	33.0	232	7.0	349 ± 95	293 ± 108	0.08 ± 0.05
	40/60	66.9	109	1.6	191 ± 37	153 ± 28	0.08 ± 0.07

^a^ The final molar fraction of *^i^* Pr‐MAC and MTC was calculated according to the NMR spectra of purified polyester copolymers. All the samples with similar *F*(*^i^* Pr‐MAC)/*F*(MTC) were grouped into one category.

^b^ Number‐average molecular weight (*M*_n_), weight‐average molecular weight (*M*_w_), and polymerization dispersity (*Ð*) were obtained by SEC measurements at a sample flow rate of 1 mL/min, with DMAc + LiCl as eluent.

^c^ Intensity‐average size (*D*_i_), number‐average size (*D*_n_), and size distribution (PDI), and the corresponding deviations were obtained by measuring the NP suspension (water) in triplicate through DLS.

**FIGURE 2:**
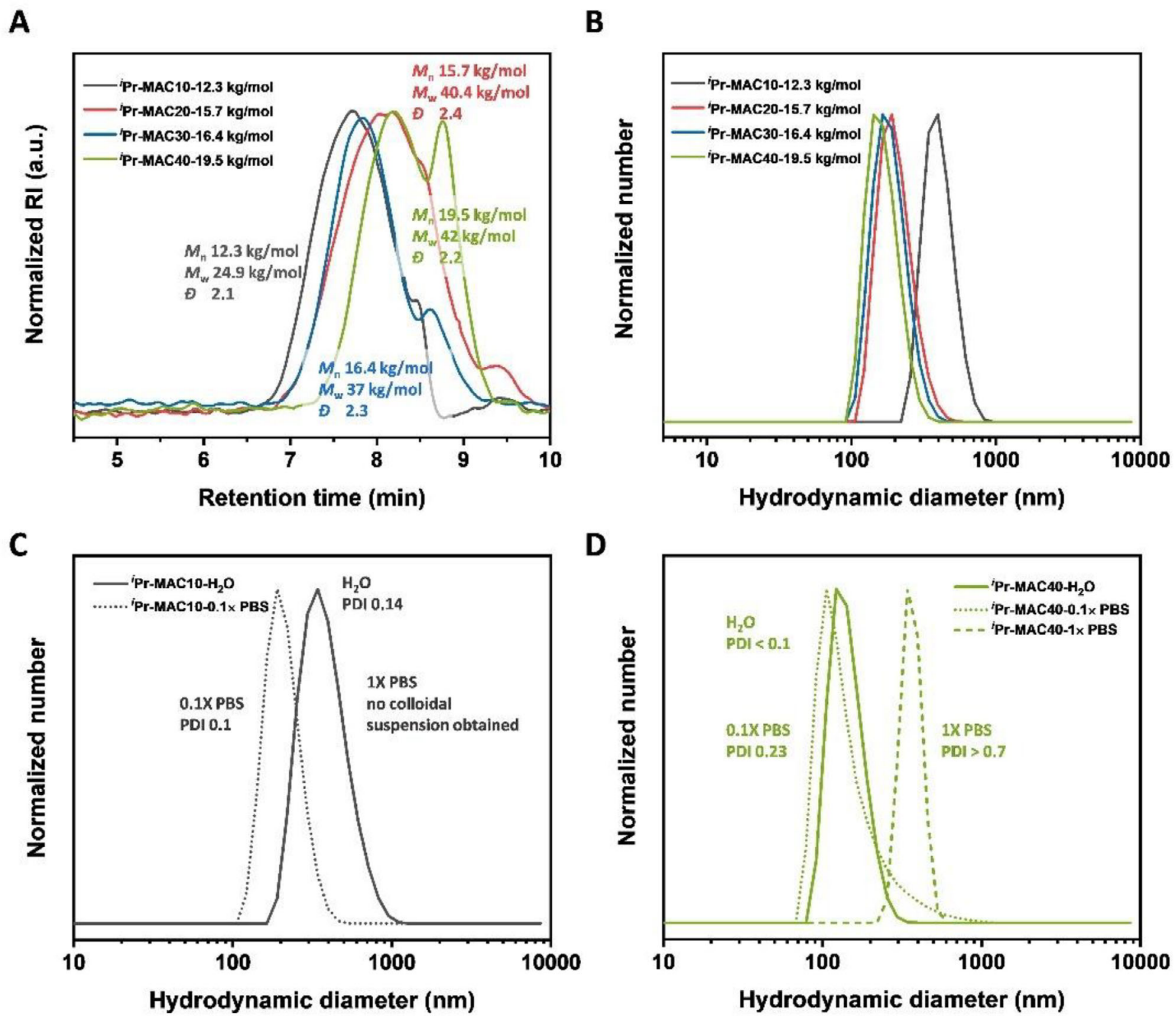
(A) Normalized refractive index (RI) chromatograms of exemplary purified polyester copolymers *^i^* Pr‐MAC10/20/30/40 with similar *M*_n_ (from 12.3 to 19.5 kg/mol), characterized by SEC measurements, and (B) the corresponding number‐size distribution curves of NPs obtained from those polyester copolymers, characterized by DLS. NPs from (C) *^i^* Pr‐MAC10 (*M*_n_ of 30.0 kg/mol) and (D) *^i^* Pr‐MAC40 (*M*_n_ of 4.5 kg/mol) were prepared in distinct aqueous media (water, 0.1×/1× PBS buffer) and the corresponding number‐size distribution curves and PDI values.

All copolymers were characterized by SEC measurements to assess their absolute molar masses (Figure 2A and Figure S10) by a light scattering detector, revealing number‐average molecular weight (*M*_n_) values from 4.5 to 66.9 kg/mol. Polyesters of all compositions contained a polymer with *M*_n_ = 12–20 kg/mol, rendering the different composition groups comparable with one another. Similar to our previous study, a greater *F*(*^i^* Pr‐MAC) leads to higher dispersity (*Ð*), rising from 1.5–2.1 for *^i^* Pr‐MAC10 to 1.6–7.0 for *^i^* Pr‐MAC40 due to the more complex polymeric structures as more *^i^* Pr‐MAC is embedded [41]. The successful polymerization of Poly(*^i^* Pr‐MAC‐co‐MTC) copolymers provides a synthetic route that complements existing methods for producing polymers such as PBAEs. This approach of polymer synthesis broadens the methodological options available. By establishing this polymerization, the study contributes to the diversity of synthetic strategies for functional polymers [42, 43, 44]. Nanoprecipitation was then performed on all copolymers to ensure that no effect of *F*(*^i^* Pr‐MAC), molar mass, or dispersity is overlooked. They were dissolved in THF as a good solvent and added dropwise into deionized water to induce NP formation by nanoprecipitation. After removing THF completely, the size distributions of the corresponding NPs were assessed by DLS (Table 1). A few general trends became visible (Figure S12). With increasing *F*(^i^Pr‐MAC) content, smaller NPs were obtained, ranging from 230–400 nm for NPs from *^i^* Pr‐MAC10, from 233–263 nm for NPs from ^*i*^Pr‐MAC30, and finally went down to 153 to 293 nm for NPs from *^i^* Pr‐MAC40. By comparing NPs composed of *^i^* Pr‐MAC10/20/30/40 with the previously noted similar *M*_n_ values of 12–20 kg/mol (Figure 2A), we confirmed this trend (Figure 2B), indicating an enhanced stability of a colloidal suspension with higher *^i^* Pr‐MAC content. Notably, all NPs showed a very small PDI (< 0.1). No correlation of the size or PDI of the NPs to the molar mass or dispersity of the polymers could be identified. A next series of nanoprecipitation was performed in phosphate buffered saline (PBS buffer) to check the applicability of the NPs in a more polar cellular microenvironment. While 1 × PBS buffer failed to produce NP suspensions, lowering the PBS buffer to 0.1 × enabled the formation of NPs with good data quality (PDI < 0.25, see Figure 2C,D).

The stability of NP suspension at room temperature showed that NPs from *^i^* Pr‐MAC30/40 exhibit stable *D*_n_ and low PDI values for at least 10 days, while NPs from *^i^* Pr‐MAC10/20 (*M*_n_ of 12.3 and 15.7 kg/mol, respectively) show a remarkable increase in *D*_n_ and PDI over 5 days (Figure 2C), possibly following ester hydrolysis. Most notably, stability in size and PDI could still be reached in spite of degrading ester units. For instance, NPs from *^i^* Pr‐MAC10 with a *M*_n_ of 49.0 kg/mol maintain stable *D*_n_ and persistently low PDI over 16 days (Figure S13), although SEC analysis reveals that hydrolysis occurred within 15 days (Figure S14).

**FIGURE 3:**
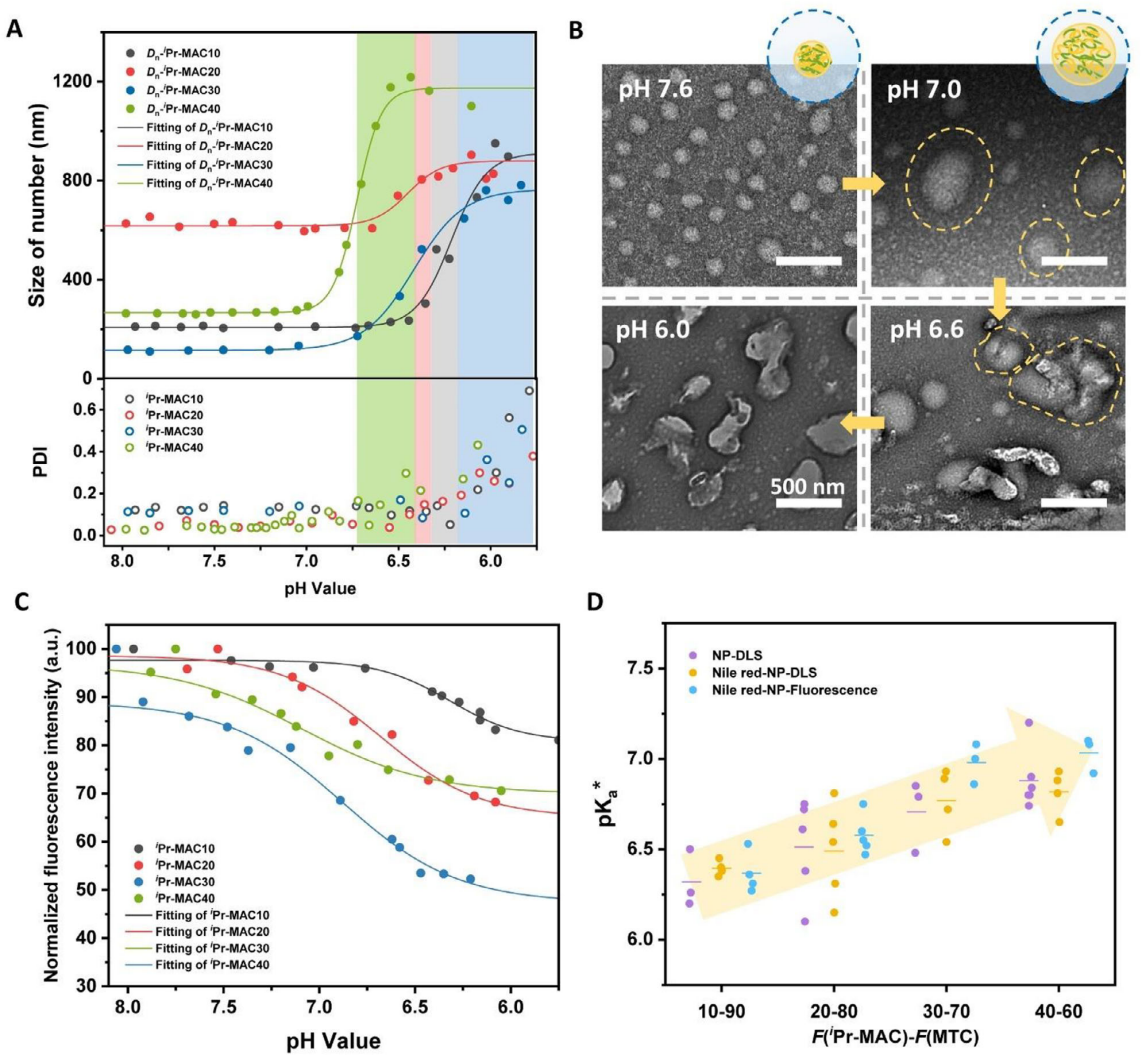
Characterization of pH‐responsiveness of NPs from *^i^* Pr‐MAC10/20/30/40 by different means. (A) The evolution of number‐average size (solid dots) and PDI (hollow dots) is characterized by SEC as a function of pH value. Note that the coloured regions indicate the pH ranges (from the pH value where the corresponding coloured region starts to the pH value where characterization ended) where ill‐shaped correlation functions were recorded. (B) TEM images of NPs from *^i^* Pr‐MAC40 (*M*_n_: 4.5 kg/mol, *Ð*: 4.5) at pH of 7.6, 7.0, 6.6, and 6.0. Scale bar: 500 nm. (C) Fluorescence intensity decline pattern of Nile‐red loaded NPs from *^i^* Pr‐MAC10/20/30/40 at varying pH values. (D) Distribution of pK_a_^*^ diagrams for NPs yielded from different *F*(*^i^* Pr‐MAC)/*F*(MTC) ratios detected through various characterization methods. Purple dots: pKa*‐D1, Yellow dots: pKa*‐D2, Blue dots: pKa*‐F.

The hydrophilic‐hydrophobic transition of these pH‐responsive polyester copolymers, as previously noted [41], may cause NP size variations in response to pH changes and hence the degree of protonation. DLS measurements were conducted to verify this behavior, and the pH of NP suspensions was altered from around 8 to 6 and plotted against *D*_n_ and PDI values to determine the characteristic half‐swelling point (pK_a_^*^‐D1, pH value at which the size reaches half of its increase). As depicted in Figure 3A, NPs initially maintain stable sizes before a decisive size increase occurs consistently across all samples and reproducibly across multiple tests when decreasing the pH (Figure S15). In contrast, control groups with constant pH show no size variation throughout the experiments (Figure S16). Moreover, NPs composed of polyester copolymers with a different molar mass but similar *F*(*^i^* Pr‐MAC)/*F*(MTC) ratio, show very similar pK_a_^*^‐D1 values. For instance, NPs from *^i^* Pr‐MAC40 show pK_a_^*^ values fluctuating only between 6.7 and 6.9, while the *M*_n_ of *^i^* Pr‐MAC40 varies from 4.5 to 66.9 kg/mol (Figure S17). Furthermore, we noticed that NPs from copolymers with a higher *F*(*^i^* Pr‐MAC) show enlarged sizes at higher environmental pH levels, indicating an earlier response to the addition of acid, following a higher possible degree of protonation. The coloured regions in Figure 3A indicate the ill‐shaped correlation functions obtained by DLS for the individual samples within specific pH ranges, alongside a surge in PDI. This stems most likely from the multitude of occurring processes that include swelling and disruption of the NPs as well as degradation and agglomeration of the individual polymer chains, resulting in samples that are not ideal for DLS.

The morphological variations of NPs at varying pH levels were further validated by TEM. NPs from *^i^* Pr‐MAC40 (*M*_n_ of 33.0 kg/mol) were analysed as an example (Figure 3B). The original NPs exhibit spherical shapes with a uniform size distribution at a pH of 7.6. As the pH dropped to 7, a noticeable increase in NP size was observed (indicated by dashed circles). Further reducing the pH to 6.6 and 6 leads to the collapse of the NP structures (indicated by dashed circles), since NPs swell and degrade at the same time. These observations go in line and hence confirm the DLS results, where we observed ill‐shaped correlation functions and a surge in PDI values stem from these mentioned processes. Nevertheless, the representative NPs demonstrate the expected swelling behavior with decreasing environmental pH, highlighting their significant potential for targeted delivery in pH‐sensitive microenvironments.

This hypothesis was next confirmed by a Nile red‐encapsulated system. Prior to fluorescence measurements, DLS characterizations on Nile red‐loaded NPs were conducted, successfully confirming that loading the NPs does not affect their pH‐responsiveness (Figures S18 and S19). These measurements resulted in our own pK_a_^*^‐D2 values, which were also recorded. Decreasing the pH values was supposed to release the encapsulated cargo. Since Nile Red shows a decrease in fluorescence when going from a hydrophobic (NP) to a hydrophilic (buffer) environment, tracking the fluorescence allowed for following the release regime. The point where half the dye was released was termed pK_a_^*^‐F (Figure 3C). In consistency with the DLS measurements, NPs from polyesters with higher *F*(*^i^* Pr‐MAC) ratios show an earlier and more pronounced decline in fluorescence intensity at elevated pH ranges. These values gradually decrease from 7 for NPs from *^i^* Pr‐MAC40 to 6.4 for those from *^i^* Pr‐MAC10. Good reproducibility of measurements can be seen, with representative NPs from *^i^* Pr‐MAC10/20/30 (*M*_n_ of 12.3, 4.6, and 25.4 kg/mol) showing similar half‐release points of around 6.3–6.4, 6.5–6.8, and 6.9–7.1, respectively (Figure S20).

A scatter plot, representing the pK_a_^*^ values of NPs at varying *F*(*^i^* Pr‐MAC)/*F*(MTC) ratios, is presented to flexibly accommodate the various influencing factors, including molar mass, dispersity, and characterization techniques (Figure 3D and data in Table S2). A tight correlation across all these analytical methods can be observed: pK_a_^*^‐D1/‐D2/‐F values rise from 6.3–6.4 to 6.8–7.0 as the *F*(*^i^* Pr‐MAC) increases from 10 mol% to 40 mol% and proves the great reliability of the data. The combination of electrostatic as well as hydrophobic interactions further enables codelivery of genetic material and small molecule drugs [58]. Covering this biomedically relevant range also highlights the applicability of these NPs at variable pH values in *vivo*.

**FIGURE 4:**
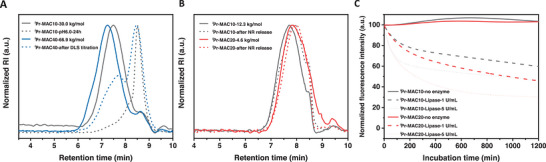
Hydrolytic degradation of polyester copolymers induced by acid characterized by SEC: (A) Normalized RI chromatograms of *^i^* Pr‐MAC10 before (*M*_n_ of 30.0 kg/mol, black solid line) and after preserving under acidic condition for 24 h (black short dashed line), *^i^* Pr‐MAC40 before (*M*_n_ of 66.9 kg/mol, blue solid line) and after subjected to pH‐dependent DLS characterization (blue short dashed line); (B) Normalized RI chromatograms of *^i^* Pr‐MAC10 before (*M*_n_ of 12.3 kg/mol, black solid line) and after subjected to Nile red‐release measurements (black short dashed line), *^i^* Pr‐MAC20 before (*M*_n_ of 4.6 kg/mol, red solid line) and after subjected to Nile red‐release measurements (red short dashed line). Enzymatic degradation of Nile red‐loaded NPs: (C) Time‐dependent normalized fluorescence intensity of Nile red‐loaded NPs composed of *^i^* Pr‐MAC10 (black lines, *M*_n_ of 12.3 kg/mol) and *^i^* Pr‐MAC20 (red lines, *M*_n_ of 4.6 kg/mol) under varying conditions: incubation in the presence of Lipase from *pseudomonas cepacian* at 1 U/mL (dashed lines) and 5 U/mL (short dashed lines) control groups without enzyme (solid lines).

Owing to the labile ester linkages in the polymer backbone, the hydrolytic degradation under various conditions was a core part of the study. Hydrolytic degradation under weak acidic conditions (pH of 6, 24 h) was hence studied on exemplary sample *^i^* Pr‐MAC10 (*M*_n_ of 30.0 kg/mol). The significant shift in the SEC chromatogram (RI trace in Figure 4A) toward higher retention time reveals the degradation and hence the great sensitivity to acid. Stability of copolymers during pH‐responsive characterizations was assessed by SEC of *^i^* Pr‐MAC40 after the pH titration of the NPs (Figure 4A) and from *^i^* Pr‐MAC10/20 after pH‐dependent fluorescence measurements (Figure 4B). The bimodal distribution or slight shift in the RI chromatograms indicates the structural degradation of ester units, implying pH‐induced degradation of all copolyesters.

The enzymatic degradation of NPs was evaluated with Lipase from *pseudomonas cepacian* (Lipase). Nile red was loaded into NPs as a fluorescent probe. An as‐prepared Nile red‐loaded NP suspension was incubated at 37 °C under enzymatic and non‐enzymatic conditions. Considering the activation of the enzyme in the buffer system, the preparation protocol for the Nile red‐loaded NP suspension was slightly modified (as detailed in the Supporting Information). As depicted in Figure 4C, when incubated without enzyme, the normalized fluorescence intensity remains nearly constant after incubation for 20 h, meaning negligible release of Nile red. Incubated with the enzyme, a remarkable reduction in fluorescence intensity is observed over incubation time, with the expected increased activity at 5 U/mL lipase compared to 1 U/mL lipase. Greater release of Nile red from NPs *^i^* Pr‐MAC20 than *^i^* Pr‐MAC10 is detected at higher lipase concentration (70 % vs 53 %), but this should not be over‐interpreted due to the low molar mass of *^i^* Pr‐MAC20 used to construct NPs (*M*_n_ of 4.6 kg/mol for *^i^* Pr‐MAC20 vs 12.3 kg/mol for *^i^* Pr‐MAC10). Similar characterizations were also performed on NPs from *^i^* Pr‐MAC30/40 (Figure S21), also showcasing great susceptibility to lipase.

**FIGURE 5:**
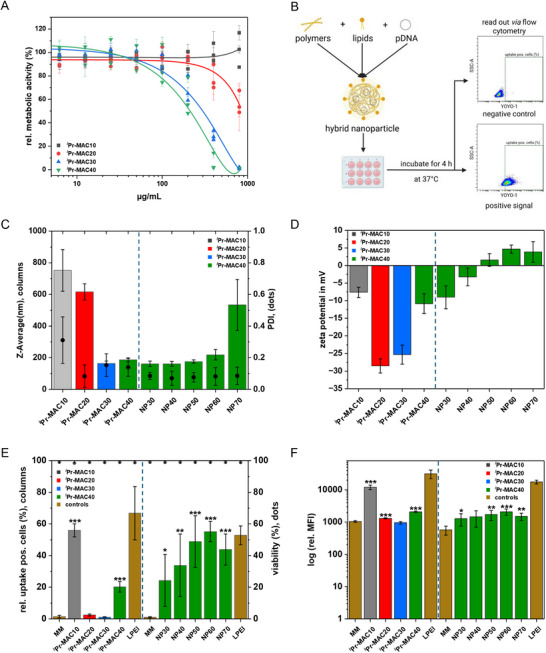
Evaluation of biocompatibility via PrestoBlue assay, size and charge via DLS, and cellular uptake efficiency. (A) Relative metabolic activity displayed at different tested polymer concentrations. (B) Illustration of hybrid nanoparticle formation and readout of the uptake efficiency assay. (C) left: DLS results at N/P 30 with different polymers tested. right: *^i^* Pr‐MAC40 at different N/P ratios. (D) left: Zeta potential of formulated particles derived from different polymers at N/P 30. right: *^i^* Pr‐MAC40 at different N/P ratios. (E) left: Relative uptake positive cells in percent after 4 h in DMEM low glucose with 10% serum + 10 mM HEPES buffer (D10H) medium at 37°C derived from the tested particles at N/P 30 and viability in %. Right: *^i^* Pr‐MAC40 at different N/P ratios. (F) left: Relative mean fluorescence intensity after 4 h in DMEM low glucose with 10% serum + 10 mM HEPES buffer (D10H) of tested polymers at N/P 30; right: *^i^* Pr‐MAC40 at N/P ratios. All: ^*^0.1, ^**^0.05, ^***^0.01 significant differences to MM control (n=3) by ANOVA.

The synthesized polymers (see Table S1) were tested for their biocompatibility and gene delivery capabilities. Evaluating biocompatibility is of major interest since polymers with a high density of protonatable amines, and therefore a high positive charge density, lead to both—a higher cytotoxicity and the ability to efficiently bind genetic material [59]. The negative effect of the high density of amines in polymers on biocompatibility has already been described in detail [60, 61, 62]. Therefore, biocompatibility has been evaluated for different synthesized polymers by using a PrestoBlue assay on L929 cells (based on ISO10993‐5) over 24 h (Figure 5A and Figure S22 of the Supporting Information) [63]. The test revealed that relative metabolic activity depends on the amount of amines in the various copolyesters (Figure 5A). At higher levels (*^i^* Pr‐MAC20‐40), there was a measurable decrease in cell viability at increased polymer concentrations. No negative influence on cell viability was measurable with *^i^* Pr‐MAC10. Overall, all polymers showed high biocompatibility up to concentrations of 100 µg/mL polymer with relative metabolic activity rates of over 70 %, at which the polymers were classified as non‐toxic, which is a remarkable achievement for amine‐bearing polymers.

Following a thorough evaluation of the polymers' biocompatibility, hybrid nanoparticles (hNP) were successfully formed by combining these polymers with the lipid excipient DMG‐PEG_2000_ as surfactant. This formulation strategy was employed to enhance particle stability, a method that has been previously described in various studies concerning the formation of hybrid nanoparticles. (Figure 5C, D) [64, 65, 66, 67]. The process of hNP formation for measuring uptake efficiency is shown in (Figure 5B and Section S4 of the Supporting Information). In contrast to covalent integration of lipids, this approach represents a versatile and highly flexible platform [68]. Gene delivery potential was evaluated using a cellular uptake assay without organic solvents using a pH shift method, where the polymers were dissolved in sodium acetate buffer (pH 5.5) and neutralized during the formulation process. This formulation method is referred to as blue formulation in the following section and has been described in detail elsewhere [69] (see Figure 5B for workflow). HNPs with a molar nitrogen‐to‐phosphate (N/P) ratio of 30 loaded with 10 µg/mL pDNA (pCMV‐M1 isolated with the EndoFree Plasmid Mega Kit) were investigated regarding size and zeta potential (Figure 5C,D, left side). The *^i^* Pr‐MAC30 and −40 polymers formed hNPs below 200 nm with PDI values below 0.2, i.e. preferred ranges for biological investigations. In contrast, copolymers with a lower amine content (*^i^* Pr‐MAC10 and 20) were unable to form particles of these quality criteria with that method. The zeta potential of all tested particles was negative, with *^i^* Pr‐MAC20 achieving the lowest value.

As *^i^* Pr‐MAC40 contains the highest number of amines, it was selected as the most promising polymer for formulating hNPs. Particles with PDI values below 0.2 were formulated at N/P ratios up to 60. Along with measuring the hydrodynamic diameter, the zeta potential was also assessed (see Figure 5C,D, right side). At low N/P ratios, the zeta potential was negative, but it shifted to positive values when the N/P ratio increased to between 40 and 50. The presence of positively charged amines was expected to result in a positive zeta potential, but this was not always the case. By neutralising the particles with tris‐buffered saline (TBS), the zeta potential might be influenced by the additional ions included in TBS. However, TBS is necessary to achieve isotonic conditions, and the effects of the surrounding buffer of nanoparticles have been described elsewhere [70].

Following the successful formulation of the NPs, the cellular uptake efficiency was evaluated. To visualize pDNA uptake in flow cytometry, YOYO‐1 iodide was used to fluorescently label the genetic material at an N/P ratio of 30 with 1 µg/mL pDNA. Linear polyethylenimine (lPEI) served as a positive control at an N/P ratio of 20 and 1 µg/mL pDNA on cells, while the master mix (MM) served as a negative control at the same amount of pDNA. The relative percentage of uptake‐positive cells, as well as cell viability, is represented in Figure 5E (left panel). The hNPs demonstrated no adverse effect on cell viability. It is noteworthy that the *^i^* Pr‐MAC10 and *^i^* Pr‐MAC40 formulations demonstrated the most significant cellular uptake, thereby underscoring their superior performance and potential. Following their biocompatibility, NPs of these RROP‐based copolymers render promising candidates for nucleic acid delivery. Although *^i^* Pr‐MAC10 demonstrated high cell uptake, its reduced amine content resulted in a substantially greater quantity of polymer reaching an N/P ratio of 30. This limitation guided the selection of *^i^* Pr‐MAC40 as the lead candidate for subsequent testing across a more extensive N/P range (refer to Figure 5E, right panel). When applied to human embryonic kidney (HEK293T) cells, *^i^* Pr‐MAC40 demonstrated an increase in uptake‐positive cells up to an N/P ratio of 60, without compromising cell viability. However, exceeding this ratio (N/P 70) resulted in larger particle sizes and decreased uptake efficiencies, likely due to size‐related barriers in cellular internalization.

This is further supported by the relative mean fluorescence intensity (MFI) data presented in Figure 5F (left panel). It was demonstrated that both *^i^* Pr‐MAC10 and *^i^* Pr‐MAC40 resulted in increased intracellular fluorescence, most likely due to expression of the previously included genes, thereby signifying their efficacy in facilitating nucleic acid delivery. For *^i^* Pr‐MAC40, the MFI increased with increasing N/P ratio, reaching a peak at N/P 60, but then dropping at an N/P ratio of 70. This is consistent with the observed decrease in uptake efficiency due to excessive particle size. In combination, the NP formation, cellular uptake, biocompatibility, and likely gene expression underline the enormous application potential of these polyesters from RROP of CKAs. Future research should focus on refining polymer design and optimizing nanoparticle formation to enhance the cellular uptake of genetic materials and achieve transfection for subsequent applications. Continued innovation in these areas will be key to unlocking the full potential of these delivery systems, paving the way for more effective gene‐based therapies.

## 3 Conclusions

Ultimately, this work systematically studied the transition points of pH‐responsive NPs prepared by nanoprecipitation of CKA‐derived polyesters. With more *^i^* Pr‐MAC embedded into the polyester backbone, the NPs exhibited greater sensitivity to acid, displaying higher transition points. A hybrid nanoparticle formulation was successfully implemented by adding the stealth‐lipid PEG‐DMG to increase particle stability and prevent aggregation. The combined data from hydrodynamic size, zeta potential, uptake efficiency, and fluorescence intensity point to *^i^* Pr‐MAC40 as the most promising polymer candidate. The optimized balance of amine content, biocompatibility, and delivery efficiency across a range of N/P ratios makes it particularly suitable for further development in nucleic acid delivery applications. Although capable of binding and delivering pDNA into target cells, the performance of these pH‐responsive polyester copolymers currently does not yet rival PEI in this application, while demonstrating their significant potential to construct nanocarriers for pH‐triggered uptake in the future. The integration of DMG‐PEG_2000_ with fully degradable, pH‐responsive, and fully biodegradable polyesters from RROP enabled stable polymeric nanoparticles with efficient cellular uptake, highlighting a novel formulation strategy. Unlike conventional polymer–lipid hybrid systems, where phospholipids form a separate layer, our approach incorporates the lipid to directly stabilize the polyester core, offering a unique pathway for biocompatible gene delivery vectors. The successful uptake of the hNP, as demonstrated in this study, is a vital step toward achieving transfection. However, the efficient release of the genetic material within the target cells needs to be optimized. Therefore, future work will concentrate on elucidating and optimizing the release mechanisms to ensure successful transfection and realize the full therapeutic potential of these nanoparticles. Thus, our findings demonstrate that the delicate balance between cationic group density, hydrophobic modifications, and polymer degradability critically governs both cytotoxicity and gene transfer performance, in particular for highly degradable polymers. Furthermore, the high concentrations of these polyester copolymers posed negligible side effects on cellular viability, showcasing their excellent biocompatibility over traditional amine‐bearing vinyl polymers. This approach led to the option of fine‐tuning nanoparticle formulations capable of transferring genetic material into cells and hence proves that amine‐bearing CKAs are suitable to push polyesters from RROP into application‐oriented research in the near future.

## 4 Experimental Section

The experimental section and supporting results can be found in the Supporting Information.

## Author Contributions

The manuscript was written by contributions from all authors and all authors gave their approval for the final version. **Yiyi Deng**: conceptualisation, formal analysis, investigation, data curation, writing – original draft, writing – review and editing, visualisation, funding acquisition. **Jonas Debbeler**: conceptualisation, formal analysis, investigation, data curation, writing – original draft, writing – review and editing, visualisation. **Anja Traeger**: conceptualisation, methodology, formal analysis, resources, data curation, writing – review and editing, supervision, project administration, funding acquisition. **Jens Gaitzsch**: conceptualisation, methodology, formal analysis, resources, data curation, writing – review and editing, supervision, project administration, funding acquisition.

## Conflicts of Interest

The authors declare no conflicts of interest.

## Supporting information




**Supporting File**: marc70088‐sup‐0001‐SuppMat.pdf.

## Data Availability

The data that support the findings of this study are available from the corresponding author upon reasonable request.
