# Supplementary material for: Degradable Nanocarriers Capable of Gene Delivery Derived from pH‐Responsive Polyester: RROP Copolymerization Between Cyclic Ketene Acetals
Source: Macromol Rapid Commun. 2025 Oct 16;47(14):e00722. doi: 10.1002/marc.202500722 (PMC13384792; doi:10.1002/marc.202500722)
Supplement: Supplementary file 1 — Supporting File: marc70088‐sup‐0001‐SuppMat.pdf. [file MARC-47-e00722-s001.pdf]

Electronic Supporting Information

Degradable Nanocarriers Capable of Gene Delivery Derived from pH-Responsive Polyester: RROP Copolymerization Between Cyclic Ketene Acetals

Yiyi Deng,[a,b,c,#] Jonas Debbeler,[d,#] Anja Traeger,[d,e,*] Jens Gaitzsch[a,*]

[a] Leibniz-Institut für Polymerforschung Dresden e.V., Hohe Straße 6, 01069 Dresden, Germany
[b] Fakultät Chemie und Lebensmittelchemie, Technische Universität Dresden, Bergstraße 66, 01069 Dresden, Germany
[c] Université Paris-Saclay, CNRS, Institut Galien Paris-Saclay, 91400 Orsay, France.
[d] Laboratory of Organic and Macromolecular Chemistry, Friedrich Schiller University Jena, Humboldtstr. 10, 07743 Jena, Germany
[e] Jena Center for Soft Matter (JCSM), Friedrich Schiller University Jena, Philosophenweg 7, 07743 Jena, Germany
[#] Y.D and J.D contributed equally to this work
* Corresponding author: anja.traeger@uni-jena.de (A.T.), gaitzsch@ipfdd.de (J.G.)

Table of Contents

Table of Contents 2
1. Materials 3
2. Characterization methods and measurements 3
3. Physicochemical characterization of hNPs 5
4. Biological characterization 6
5. Statistical analysis 7
6. CKA synthesis 8
7. Copolymerization of ^{i}Pr-MAC and MTC 12
8. 2D NMR characterizations on nanoparticles from ^{i}Pr-MAC30 17
9. SEC characterizations on ^{i}Pr-MAC10/20/30/40 20
10. DOSY characterization on ^{i}Pr-MAC30 21
11. DLS characterizations on nanoparticles 22
12. Colloidal suspension stability characterized by DLS and SEC 23
13. pH-Responsiveness of nanoparticles characterized by DLS measurements 25
14. pH-Triggered Nile red release characterized by fluorescence spectroscopy 30
15. Enzymatic degradation characterized by Tecan plate reader 31
16. Cellular uptake and gene expression 32

1. Materials

Diethylene glycol (ReagentPlus grade, 99.0%), chloracetaldehyde dimethylacetal (≥ 99.0%, GC), DOWEX (hydrogen form, 100-200 mesh), *tert*-butanol (ACS reagent, ≥ 99.0%), potassium *tert*-butoxide (ACS reagent, ≥ 98.0%), diethanolamine (ACS reagent, ≥ 98.0%), 2-bromopropane (99.0%), ethyl chloroformate (≥ 98%, GC), triethylamine (≥ 99.5%, GC), aluminum chloride (NH_4Cl, reagent grade, 98%), bis(cyclopentadienyl)titanium (IV)) dichloride (98.0%), methylmagnesium chloride solution (3M in THF) and potassium hydroxide (ACS reagent, 90%, ≥ 85%, pellets) were purchased from Sigma Aldrich and directly used without further purification. 2,2′-Azobis(2-methylpropionitrile) (AIBN, 98.0%) was purchased from Sigma Aldrich and re-crystallized in methanol for purification before usage. Sodium sulfate (Na_2SO_4, ACS reagent, ≥ 99.0%), magnesium sulfate ($MgSO_4$, ACS reagent, ≥ 99.0%) and sodium chloride (NaCl, ACS reagent, ≥ 99.5%) were purchased from Honeywell Fluka. Diethyl ether (99.5%) was purchased from CHEMSOLUTE. Methanol (99.5%), anhydrous tetrahydrofuran (THF, 99.5%), dichloromethane (> 99.8%) and chloroform (99.0%) were purchased from Acros Organics. Ethyl acetate (Analytical reagent grade, ≥ 99.8%) was purchased from Fischer Scientific. N-hexane (99.0%) was purchased from Supelco. Toluene (> 99.8%) was purchased from Thermo Scientific. Pre-treated RC dialysis membrane (MWCO: 1 k) was purchased from Carl Roth. PrestoBlue cell viability reagent (Thermo Fisher Scientific). Sodium Acetate Buffer (3 M), pH 5.5, RNase-free and Invitrogen™ UltraPure™ DNase/RNase-Free Distilled Water were purchased from Thermo Fisher Scientific. DMG-PEG_{2000} has been purchased from Avanti Polar lipids. YOYO-1 iodide has been purchased from Invitrogen (Y3601). 10× concentrated TRIS buffered saline has been purchased from Thermo Scientific (J62938.K2). Dulbecco's modified eagle medium (DMEM), 1 M HEPES, Dulbecco’s PBS (1×), without Ca & Mg, without Phenol Red and trypsin/EDTA solution have been purchased from Capricorn Scientific. HEK-293-T cells (human embryonic female kidney cells) were purchased from DSMZ (ACC 635) and used up to passage number 25. pCMV-M1 was a gift from Linda Wordeman (Addgene plasmid # 23007; http://n2t.net/addgene:23007; RRID:Addgene_23007).[1] Endofree Plasmid Mega Kit has been purchased from Qiagen.

2. Characterization methods and measurements

Nuclear magnetic resonance spectroscopy (NMR). NMR spectra were acquired at a Bruker Avance III 500 MHz spectrometer with a frequency of 500 MHz for ^{1}H NMR and 125 MHz for ^{13}C NMR, and results were recorded at room temperature.

2D NMR techniques, including DOSY, HSQC, HMBC and COSY measurements on polyester copolymers ^{i}Pr-MAC30 were performed on a Bruker 400 MHz spectrometer at room temperature. $CDCl_3$ was used as solvent.

Size-exclusion chromatography (SEC). SEC measurements were conducted at 25 ˚C with a PolarGel-M column (300 × 7.5 mm), using HPLC-Pump 1200 pump system (Aligent technologies) equipped with a multi angle laser light scattering (MALLS) detector (MiniDAWN TREOS II, Wyatt Technology) and a RI detector (K-2301 from KNAUER). DMAc with 3 g/L LiCl was used as an eluent and the flow rate was 1 mL/min. Before measurements, well-dissolved solution was filtered with a 0.2 µm PTFE filter. All the copolymers were measured for two times.

To evaluate possible degradation after pH-responsive characterization, the organic compounds of nanoparticles were extracted with ethyl acetate. After removing organic solvent completely, the residuals underwent SEC measurements.

Dynamic light scattering (DLS). The size distributions of nanoparticle suspension prepared in water and PBS buffer, and their size variations against pH value were recorded by DLS measurements through a Zetasizer Nano-series instrument (Malvern Instruments, UK). DLS measurements were performed at 25 °C and data were collected by NIBS method (non-invasive back-scatter) with a helium–neon laser (4 mW, l = 632.8 nm) at fixed scattering angle of 173°. Specifically, when performing pH-dependent size measurements, the pH value of nanoparticle suspension was altered by adding 0.01 M NaOH and HCl and the pH value was recorded by a pH meter.

Transmission Electron Microscopy (TEM). Before sample preparation, copper grids (400 mesh) were subjected to plasma treatment at a power of 70 W for 20s. Aliquots of 10 µL of diluted nanoparticle suspensions (^{i}Pr-MAC40, 0.25 mg/mL) were deposited for 2 min on copper grids covered with Formvar-carbon film. The excess solution was removed with a filter paper and then 3 µL of uranyl acetate (2%, w/v) was added on the copper grids and the staining process lasted for 1 min. Excess of uranyl acetate was blotted off using a filter paper. The morphologies of nanoparticles were measured by Libra@120. To validate the morphological variations of nanoparticles at different pH levels, the pH of nanoparticle suspension was altered by adding 0.01 M HCl, and the nanoparticle suspension was subjected to sample preparation promptly.

Fluorescence spectroscopy. The Nile red-release was evaluated by fluorescence spectroscopy. Specifically, 0.05 mg/mL Nile red/THF stock solution was added into the polyester copolymer/THF solution during nanoprecipitation to prepare Nile red-loaded nanoparticles. The final Nile red concentration was around 0.0005 mg/mL, and the copolymer concentration was around 0.5 mg/mL. The pH of nanoparticle suspension was altered by adding 0.01 M NaOH and HCl solution, and the pH-dependent fluorescence intensity was acquired on a Perkin Elmer LS55 fluorescence spectrometer

using a quartz cell at room temperature. The excitation/emission wavelengths (λ_{ex} /λ_{em}) were set up as 550 and 630 nm with a bandwidth of 3 nm.

Enzymatic degradation. To evaluate the enzymatic degradability of the resulting nanoparticles, Nile red was used as a fluorescent probe. Specifically, a 0.05 mg/mL Nile red/THF stock solution was added to the polyester copolymer/THF solution during nanoprecipitation to prepare Nile red-loaded nanoparticles. Lipase from *pseudomonas cepacian* was dissolved into 1× PBS buffer to prepare fresh Lipase/PBS stock solution. Next, 0.2 mL Lipase/PBS stock (10 U/mL and 50 U/mL) solution was mixed up with 1.8 mL Nile red-loaded nanoparticles suspension to achieve a final enzymatic concentration of 1 U/mL and 5 U/mL, and a final PBS buffer concentration of 0.1×. The Lipase/Nile red loaded-nanoparticle suspension was then pipetted into a 96-well plate and incubated at 37 °C. The control groups, in which no enzyme was present, were incubated at 37 °C for comparison. The fluorescence intensity was detected at excitation/emission wavelengths (λ_{ex} /λ_{em}) of 550 and 630 nm over 20 h.

3. Formulation and physicochemical characterization of hNPs

Formulation of hybrid particles (hNP)

For particle preparation, polymers (^{i}Pr-MAC10, ^{i}Pr-MAC20, ^{i}Pr-MAC30 and ^{i}Pr-MAC40) were dissolved in 0.1 M sodium acetate buffer pH 5.5. Furthermore, the stealth lipid DMG-PEG$_{2000}$ has been diluted to 0.1 mg/mL in water. For hybrid nanoparticle formulation 3 mol% of DMG-PEG$_{2000}$ has been used per polymer. The required amount of DMG-PEG2000 was added to a vial and diluted with water. Polymer was added and immediately vortexed for 10 sec. For the final formulation of hybrid nanoparticles, the plasmid DNA pCMV-M1 has been diluted in water (mastermix (MM)), which served as a negative control. For the samples that were measured on their uptake efficiency, YOYO-1 has been added to the MM. In the next step MM has been applied to the polymer solution which has been vortexed for 15 s and afterwards stored for 15 min at room temperature (RT) in the dark. This step has been prepared once for hybrid nanoparticles containing the negative control MM and once with the MM with YOYO-1 dye. In the last step hybrid nanoparticles were neutralized with five times concentrated Tris buffered saline (TBS). The final amount of TBS in the particle solution was 1× concentrated, while the amount of genetic material in particle solution was 10 µg/mL. The amount of polymer was determined according to the N/P ratio of 30.

Size and size distribution

Dynamic light scattering (DLS Zetasizer Nano ZS, Malvern Instruments, Germany) was applied to determine the hydrodynamic diameter and the distribution of the polyplexes formed. 100 µL of

polyplexes of pCMV-M1 plasmid was used. DLS was conducted using DLS Zetasizer Nano ZS, Malvern Instruments, Germany with a He−Ne laser operating at a wavelength of 633 nm. After an equilibration time of 30 s, each sample was measured at 25 °C, 5 size runs were performed with 30 s per run. The counts were detected at an angle of 173°. The mean particle size was determined by the effective diameter (z-average), while the polydispersity index (PDI) of the particles was described by the distribution width. As analysis model, general purpose was used. The data was analyzed using ZS Xplorer software.

Zeta Potential

Zeta potential of the samples was measured in triplicates at 25 °C and 40 mV with measurement duration set to automatic (10 - 20 runs) after an equilibration time of 30 s and with a delay of 30 s between each measurement.

4. Biological characterization

Viability determination

The viability was investigated via PrestoBlue™ assay at Tecan infinite M200Pro multiplate reader, according to ISO-guideline10993-5 (6 replicates each day, 3 day measurements). For this purpose, L929 was seeded in a 96-well plate one day before treatment to ensure cell attachment. Cell cultivation took place at 37 °C in a humidified 5% (v/v) CO_2 atmosphere. The outer wells were excluded. After 24 h of preincubation, the used medium was replaced by 100 µL of a solution containing the polymers dissolved and diluted in Dulbecco's modified eagle medium (DMEM) plus 10% fetal bovine serum (D10) medium at the targeted concentration. 24 h later, the medium was replaced by a 10 % (v/v) PrestoBlue solution in fresh culture medium, prepared according to the manufacturer's instructions. The cells were further incubated for 45 min before fluorescence was measured at λ_{Ex} = 560/λ_{Em} = 590 nm. Non treated control cells were referred to as 100% viability, and values lower than 70% were regarded as cytotoxic. The relative percentage of viable cells was calculated as follows:

$$\text{viability (\%)}=\frac{FI_{Sample}-FI_0}{FI_{Ctrl}-FI_0}\times 100\ \%$$

FI_{Sample}, FI_0, and FI_{Ctrl} represent the fluorescence intensity of a given sample, medium without cells (the blank), and non-treated control (100% viability), respectively.

Uptake studies. The polymers tested for the uptake studies claimed in the following as (^{i}Pr-MAC10, ^{i}Pr-MAC20, ^{i}Pr-MAC30 and ^{i}Pr-MAC40) are listed in Table S1.

Table S1: Characteristics of the polymers used for biological assays

	F(MAC)/*F*(MTC)[a]	M_n (kg/mol)[b]	M_w (kg/mol)[b]	*Đ*[b]

^{i}Pr-MAC10	10/90	30.0	63	2.1
^{i}Pr-MAC20	21/79	8.6	16.1	1.9
^{i}Pr-MAC30	35/65	49.5	187	3.8
^{i}Pr-MAC40	40/60	23.3	42.7	1.8

[a] Final molar fraction of ^{i}Pr-MAC and MTC was calculated according to the NMR spectra of purified polyester copolymers; [b] Number-average molecular weight (M_n), weight-average molecular weight (M_w), and polymerization dispersity ($Đ$) were obtained by SEC measurements at a sample flow rate of 1 mL/min, with DMAc + LiCl as eluent.

For uptake studies HEK-293-T cells were seeded at 0.1×10^6 and incubated 24 h prior to treatment in D10 medium containing 10 mM HEPES buffer solution (D10H) in a 24-well plate. 1 h prior to treatment medium was replaced by fresh D10H. Besides, for particle formulation 0.5 mM of YOYO-1 solution has been used to stain 6 µg of genetic material (pCMV-M1 plasmid). The particles were added to HEK-293-T cells leading to a final concentration of genetic material of 1 µg/mL per 24 well plate. Linear polyethylenimine (lPEI) served as a positive control at a N/P-ratio of 20 and 1 µg/mL genetic material on the cells. The experiment has been performed as well by using polymer ^{i}Pr-MAC40 at the different N/P-ratios 30, 40, 50, 60, 70 and a constant concentration of genetic material of 1 µg/mL on the cells. After incubation for 4 h, cells were washed once with 500 µL of prewarmed PBS, followed by 150 µL Trypsin-EDTA for 5 minutes at 37 °C. Afterwards, the cells were resuspended in 350 µL of PBS. Resuspended cells were transferred to a 96-well plate and measured via flow cytometry. The YOYO-1 signal was measured via flow cytometry (Cytoflex LX, Beckman Coulter) at a λ_{Ex} = 488 nm and measurement at 510/20 nm Bandpass with OD1 Filter (Beckman Coulter). Viable single cells were gated according to the forward scatter/side scatter (FSC/SSC) pattern of the untreated control. YOYO-1 positive cells were gated to the master mix. (Figure S1) A minimum of 10,000 single cells were analysed. The relative mean fluorescence and relative positive cells (%) was calculated to the single cells of negative control hybrid nanoparticles containing genetic material without YOYO-1.

$$rel.\,uptake\ pos.\,cells = YOYO\ hybrid\ nanoparticles - neg.\,hybrid\ nanoparticles$$

5. Statistical analysis

All biological investigations were conducted at least in triplicate, and data were reported as mean ± standard deviation (SD). Statistical analyses were calculated using OriginPro2022b software. The one-way analysis of variance (ANOVA) was applied to determine the statistical significance. Statistical significance was notated as $*p \leq 0.05$, $**p \leq 0.01$, and $***p < 0.001$.

6. CKA synthesis

Preparation of 2-methylene-1,3,6-dioxazocane (MTC)

Diethylene glycol (70 mL, 739 mmol, 1.0 eq.), chloracetaldehyde dimethylacetal (94 mL, 826 mmol, 1.1 eq.) and DOWEX (1.01 g) were mixed in a two-necked flask and degassed with nitrogen for 5 min. The reaction mixture was stirred at 120 °C under the protection of nitrogen and the methanol formed during the reaction was removed constantly through distillation. The reaction was stopped when no more methanol was formed (after about 2 h). The crude product was subsequently purified by distillation (75 - 80 °C, 3 mbar). The whitish solid aimed product 2-(chloromethyl)-1,3,6-trioxocane was obtained (76.5 g, 459 mmol, 62%).

^{1}H-NMR (Cl-MTC, 500 MHz, $CDCl_3$) δ (ppm) 4.79 (t, *J* = 5.4 Hz, 1H, OCHO), 3.96 (m, 4H, OCH_2), 3.70 (m, 4H, OCH_2), 3.47 (d, *J* = 6.4 Hz, 2H, CH_2Cl).

2-(Chloromethyl)-1,3,6-trioxocane (20.0 g, 120 mmol, 1.0 eq.) and *tert*-butanol (HO^{t}Bu, 61.6 mL) were charged in a Schlenk tube and degassed with nitrogen for 15 min. Potassium *tert*-butoxide (KO^{t}Bu, 16.16 g, 144 mmol, 1.2 eq.) was slowly added while stirring. The mixture was stirred at 120 °C for 16 h. The reaction mixture was washed with diethyl ether and the precipitated KCl salt was filtered off with a Büchner funnel. The solvent was removed through rotary evaporator. Then the crude product was purified by 3 successive distillations (70 °C, 20 mbar). 2-Methylene-1,3,6-trioxocane (MTC) was obtained as a colorless liquid (53%).

^{1}H-NMR (MTC, 500 MHz, $CDCl_3$) δ (ppm) 4.06 (t, *J* = 4.8 Hz, 4H, OCH_2), 3.77 (t, *J* = 4.8 Hz, 4H, OCH_2), 3.67 (s, 2H, C-CH_2).

^{13}C-NMR (MTC, 125 MHz, $CDCl_3$) δ (ppm) 164.03 (1C, $\underline{C}CH_2$), 70.99 (1C, $C\underline{C}H_2$), 70.50 (2C, OCH_2), 70.40 (2C, OCH_2).

Preparation of 6-(*iso*-propyl)-2-methylene-1,3,6-dioxazocane (^{i}Pr-MAC)

Diethanolamine (8.8 g, 1 eq.) and 2-bromopropane (11.3 g, 1.1 eq.) were charged into a three-neck round bottom flask, equipped with a stir bar. K_2CO_3/water (1 g/5 mL) was introduced, and the system was sealed and flushed with argon for 30 minutes. The mixture was heated to 75 °C for 5 days. The reaction turned yellow, and then KOH/water (5 g/10 mL) was added. The mixture was dried under reduced pressure, and methanol was added, resulting in white precipitates, which were filtered out. Methanol was then removed completely by rotary evaporator, and the residuals were subsequently washed by toluene (2 mL × 2 times). The toluene phases were disposed, and then the aimed product was extracted with chloroform or dichloromethane for three times (30 mL chloroform or dichloromethane each time). The organic phase was collected and removed under reduced pressure. After drying in the vacuum oven overnight, the target product *iso*-propyl-diethanolamine was obtained as yellow oil (yield 57 %).

^{1}H-NMR (diol, 500 MHz, $CDCl_3$) δ (ppm) 3.53 (t, J = 5.4 Hz, 4H, OCH_2), 2.94 (m, 1H, NCH), 2.58 (t, J = 5.4 Hz, 4H, NCH_2), 0.99 (d, J = 6.6 Hz, 6H, N(CH)-CH_3).

Iso-propyl-diethanolamine (4.47 g, 1 eq.) was dissolved in 50 mL anhydrous THF and then the mixture was flushed with argon, cooled in an ice bath with stirring for 30 min. Ethyl chloroformate (11.6 mL, 4 eq.) was added into the flask and kept stirring for another 30 min. Subsequently, TEA (17 mL, 4 eq.) dried over Na_2SO_4 was added into the flask using a syringe pump and the feeding speed of TEA was 200 µL/min. The mixture was stirred in ice bath for 2 h and then another 17 h at room temperature. The precipitations were filtered off and then the organic phase was concentrated and subjected to column chromatography (n-Hexane/EtOAc = 3/1). Aimed substance was obtained as yellow oil with a yield from 35 to 40 %

^{1}H-NMR (carbonate, 500 MHz, $CDCl_3$) δ (ppm) 4.16 (t, J = 5.4 Hz, 4H, OCH_2), 2.94 (m, 1H, NCH), 2.73 (t, J = 5.0 Hz, 4H, NCH_2), 1.02 (d, J = 6.6 Hz, 6H, N(CH)-CH_3).

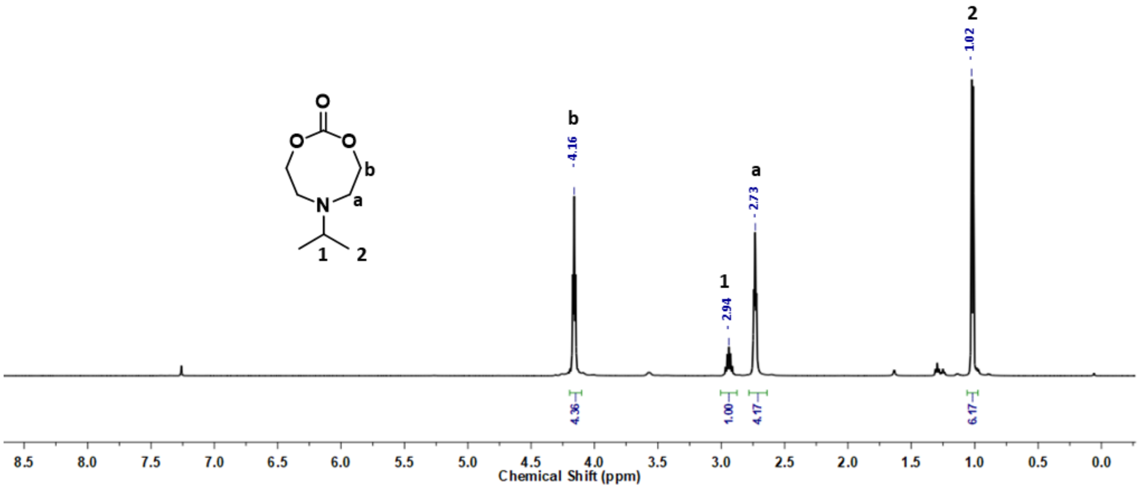

Figure S1. ^{1}H spectrum for intermediate carbonate and the signal assignments.

In a round-bottle flask, bis(cyclopentadienyl)titanium (IV)) dichloride (16.0 g, 64.3 mmol, 1.0 eq.) and 160 mL toluene were added and well mixed in the NaCl/ice bath. After stirring for 30 min, 50 mL MeMgCl in THF (150 mmol, 2.3 eq.) was added into the flask drop-wise in 30 min. The flask was kept around -10 ˚C and stirring for 1h. 6 % aq. NH_4Cl solution (2.8 g NH_4Cl/44 mL H_2O) was well-stirred in advance and cooled in the ice bath. Then, the viscous mixture was quenched with NH_4Cl solution. The mixture was stirred for another 1h. Subsequently, the mixture was washed with 60 mL cold water for 3 times and followed by 60 mL cold saturated NaCl solution for 2 times. The orange organic phase was collected and dried with $NaSO_4$. The solid content in the THF/toluene was around 7 mol%.

^{1}H-NMR (500 MHz, $CDCl_3$) δ (ppm) 0.01 (s, 6H, CH_3), 6.11 (d, 10H, CH on Cp ring).

Intermediate carbonate (2 g, 1.0 eq.), freshly prepared Petasis reagent (2.3 eq.) and 35 mL anhydrous THF were charged into a round-bottom flask and heated up to 65 ˚C. After 22 h, 200 mL *n*-hexane was added into the flask. Yellow precipitation was afforded immediately. The precipitation was isolated using filtration paper and then the solvent was removed using rotary evaporator. The concentrated mixture was diluted with *n*-hexane again and followed by the filtration step. Repeating above steps for 3 times, the concentrated viscous mixture was subjected to vacuum distillation in an oil bath afterward. Under 3 mbar, 40-65 ˚C, orange oily liquid was collected as aimed substance (yield from 11 to 30 %).

^{1}H-NMR (^{i}Pr-MAC, 500 MHz, $CDCl_3$) δ (ppm) 3.97 (t, J = 5.0 Hz, 4H, OCH_2), 3.55 (s, 2H, CCH_2), 2.92 (m, 1H, NCH), 2.71 (t, J = 5.4 Hz, 4H, NCH_2), 0.98 (d, J = 6.6 Hz, 6H, N(CH)-CH_3).

^{13}C-NMR (^{i}Pr-MAC, 125 MHz, $CDCl_3$) δ (ppm) 164.98 (1C, $\underline{C}O$) 71.41 (2C, $O\underline{C}H_2$), 69.72 (1C, $C\underline{C}H_2$), 55.56 (1C, $N\underline{C}H$), 51.68 (2C, $N\underline{C}H_2$), 19.84 (2C, N(CH)-$\underline{C}H_3$).

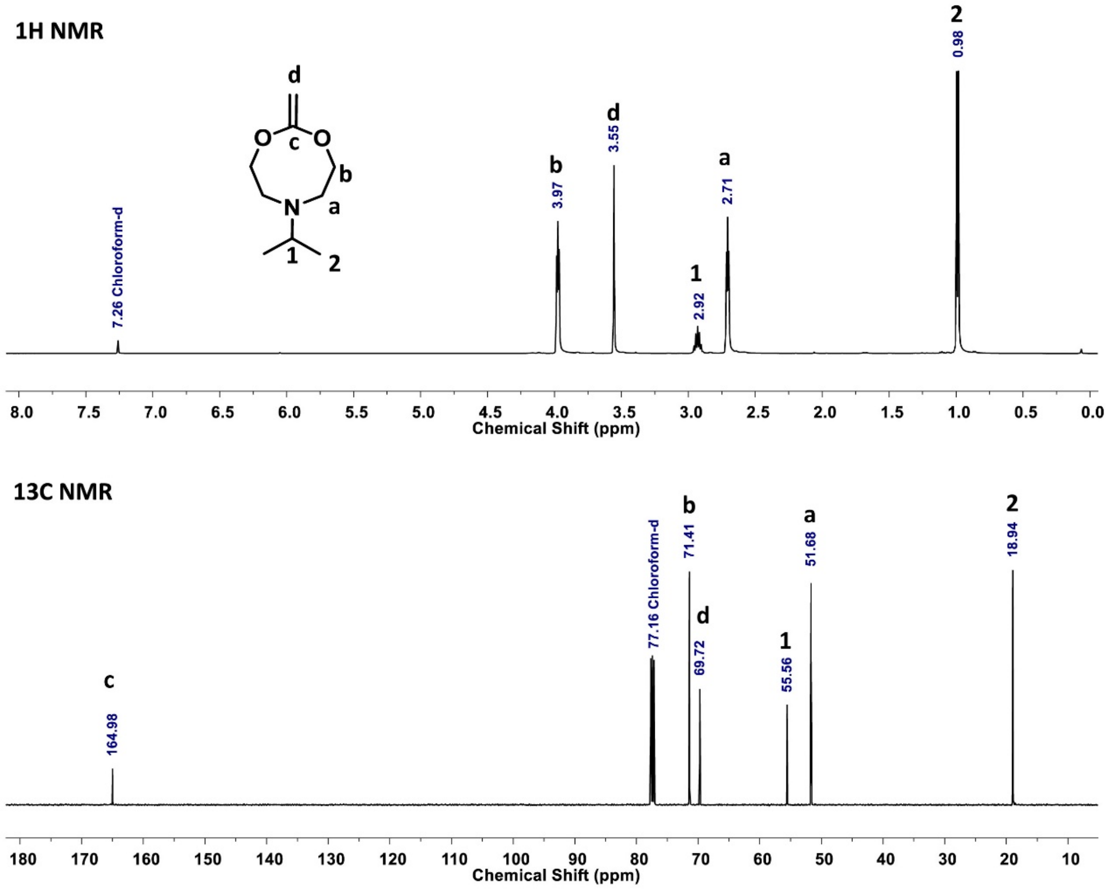

Figure S2. ^{1}H and ^{13}C NMR spectra for amine-bearing CKA 6-(*iso*-propyl)-2-methylene-1,3,6-dioxazocane (^{i}Pr-MAC) and the signal assignments.

7. Copolymerization of ^{i}Pr-MAC and MTC

In a clean glass vial, ^{i}Pr-MAC, MTC, AIBN and a small stir bar were added to perform copolymerization. The target molar fraction of ^{i}Pr-MAC in the resulting copolymer was designed varying from 10 mol% to 40 mol%. The polymerization parameters of copolymers listed in this study are recorded in **Table S1**. The reaction mixture was flushed with argon for 30 min and then heated up in an oil bath for 24 h. After polymerization, the reaction mixture became viscous and the color became darker. Diluted with some chloroform, the crude product was purified through dialysis in chloroform for 4 times and RC dialysis membrane was utilized for purification (MWCO: 1 k). Then the purified polymer was collected under reduced pressure.

The calculation of conversion of ^{i}Pr-MAC/MTC, and final molar fraction of ^{i}Pr-MAC/MTC followed the equation published earlier, and the calculated values are listed in **Table S1**:

$$\text{Conversion of MTC} = \frac{I(4.20\ \text{ppm})}{\frac{I(4.06\ \text{ppm})}{2} + I(4.20\ \text{ppm})} \times 100\ \%$$

$$\text{Conversion of } i\text{Pr-MAC} = 100\ \% - \frac{I(3.55\ \text{ppm})}{2 \times I(2.91\ \text{ppm})} \times 100\ \%$$

$$F_{\mathbf{MAC}} = \frac{I(2.91\ \text{ppm})}{\frac{I(4.20\ \text{ppm})}{2} + I(2.91\ \text{ppm})} \times 100\ \%$$

$$F(\text{MTC}) = 100\ \% - F(^{i}\text{Pr-MAC})$$

Table S1. Polymerization conditions to yield poly(^{i}Pr-MAC-co-MTC) copolymers with distinct chemical compositions and molar masses.

	Entry	f(MAC)/f(MTC)	AIBN (mol%)	% Conversion of MAC/MTC	F(MAC)/F(MTC)	M_n (kg/mol)	M_w (kg/mol)	M_w/M_n
^{i}Pr-MAC10	1	9/91	0.5	-	11/89	12.3	24.9	2.1
	2	8/92	0.65	50/17	9/91	30.0	63.0	2.1
	3	9/91	0.5	11/11	13/97	49.0	73.8	1.5
^{i}Pr-MAC20	4	15/85	0.5	50/24	24/76	4.6	14.5	3.2
	5	13/87	0.6	50/22	18/82	15.7	40.4	2.4
^{i}Pr-MAC30	6	25/75	0.5	20/13	31/69	16.4	37.0	2.3
	7	20/80	0.65	73/70	32/68	25.4	46.5	1.8
^{i}Pr-MAC40	8	25/75	0.75	-	42/58	4.5	20.2	4.5
	9	25/75	1	77/25	44/56	19.5	42.0	2.2
	10	20/80	0.65	31/7	39/61	33	232	7
	11	25/75	0.5	17/3	40/60	66.9	109	1.6

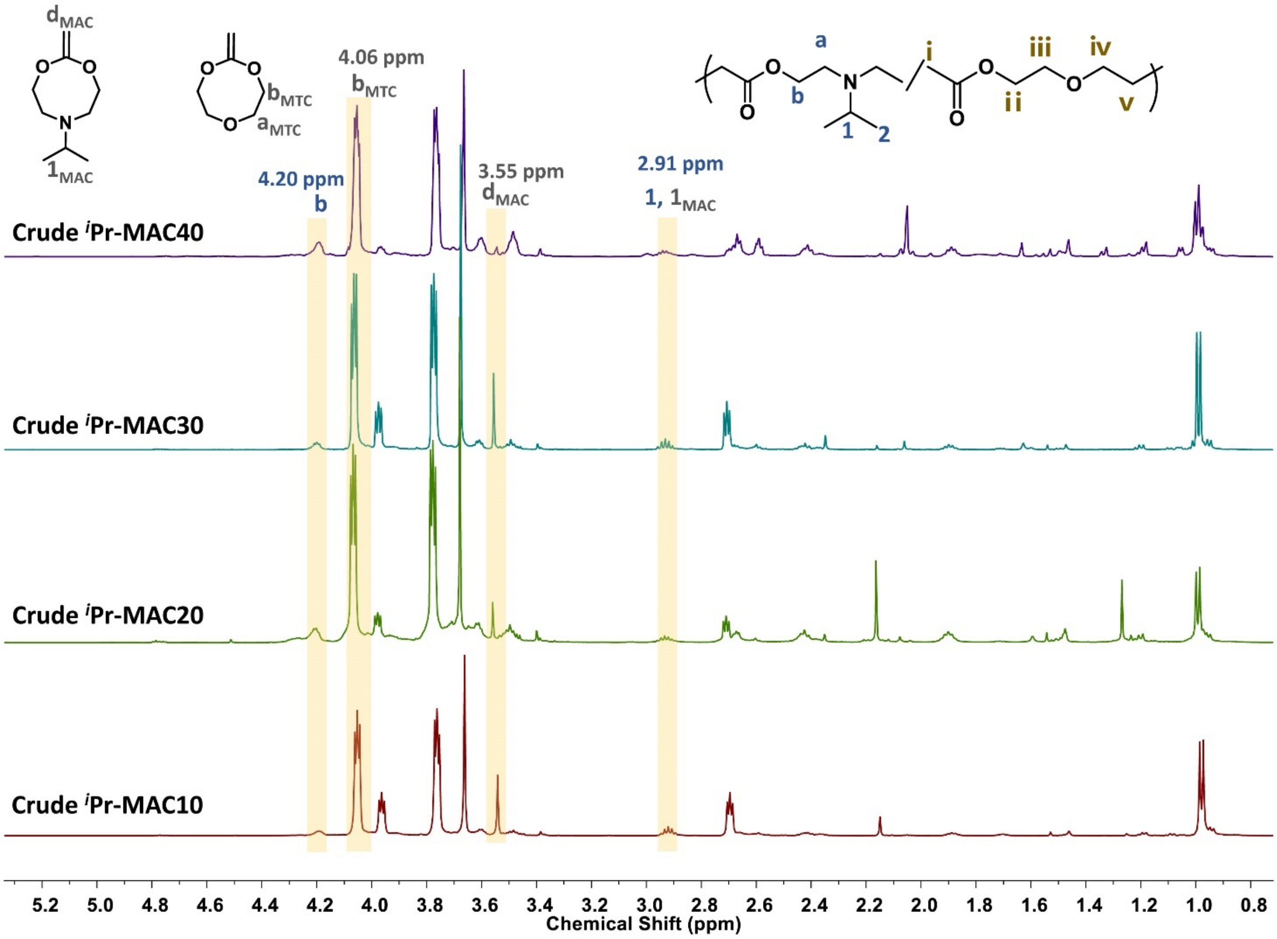

Figure S3. ^{1}H NMR spectra for crude products of exemplary polyester copolymers ^{i}Pr-MAC10/20/30/40. Resonances ascribed to protons **b** at 4.20 ppm and $\mathbf{b_{MTC}}$ at 4.06 ppm are used to calculate the conversion of MTC. Resonances ascribed to protons $\mathbf{1/1_{MAC}}$ at 2.91 ppm and $\mathbf{d_{MAC}}$ at 3.55 ppm are used to calculate the conversion of ^{i}Pr-MAC.

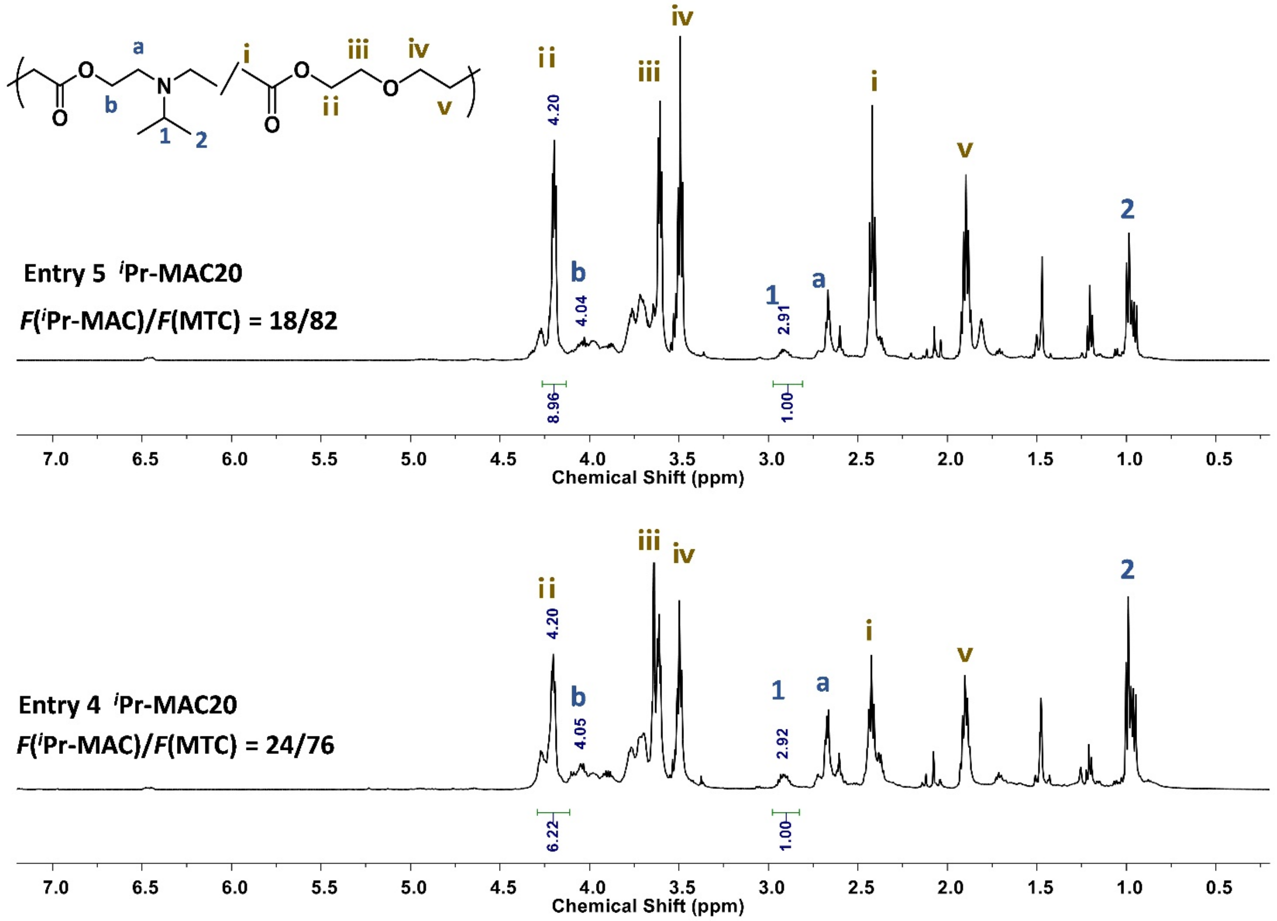

Figure S4. NMR spectra of purified polyester copolymers with slightly different F(^{i}Pr-MAC)/F(MTC)s, prepared at identical feed ratio of ^{i}Pr-MAC and MTC (f(^{i}Pr-MAC)/f(MTC) = 18 mol%/82 mol%).

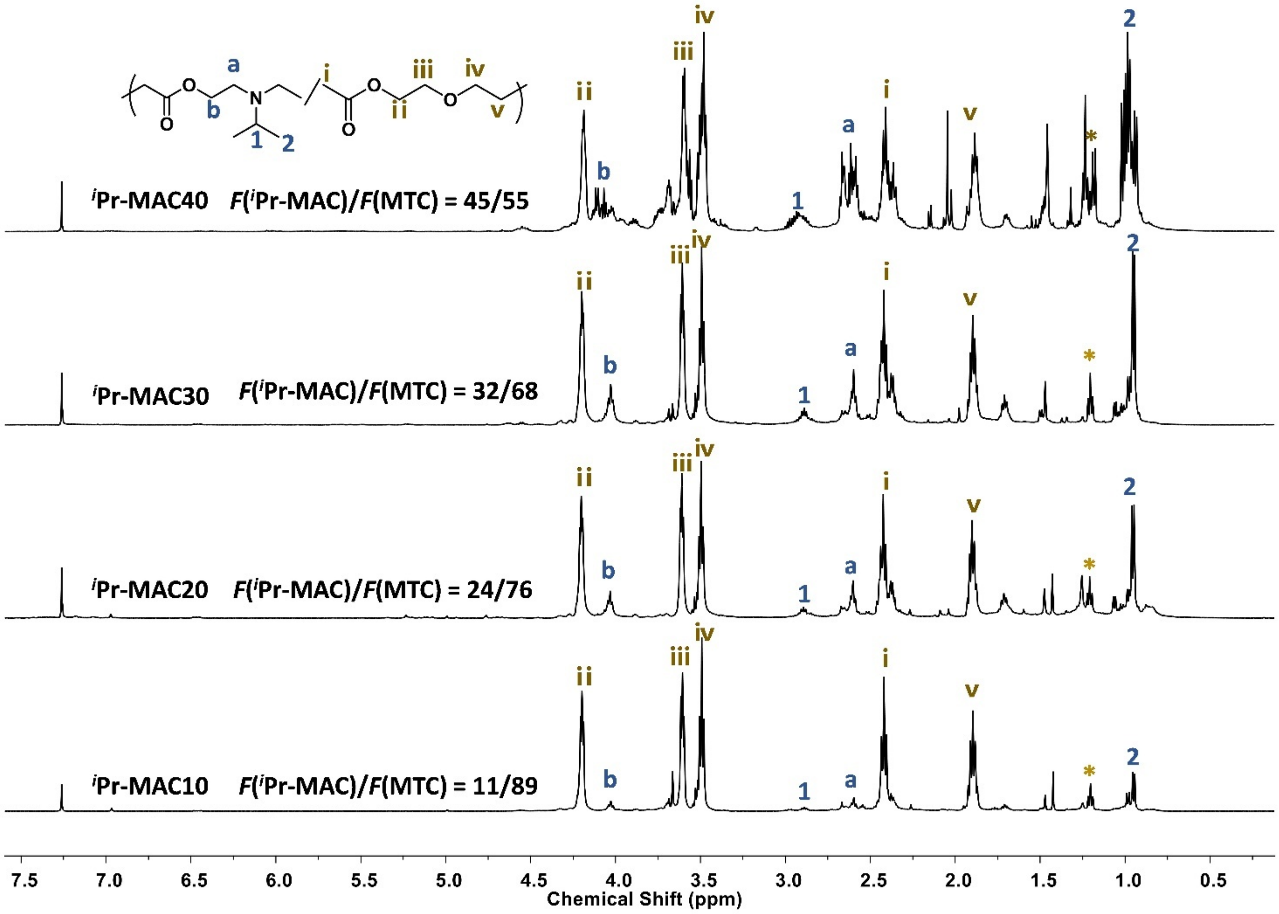

Figure S5. ^{1}H NMR spectra of purified exemplary polyester copolymers with distinct *F*(^{i}Pr-MAC)/*F*(MTC)s ranging from 11/89 to 45/55 (mol/mol). Resonances **i-v** and * related to poly-MTC segments and branching units are fully assigned, and the characteristic signals **1**, **2**, **a**, and **b** are attributed to ^{i}Pr-MAC motifs.

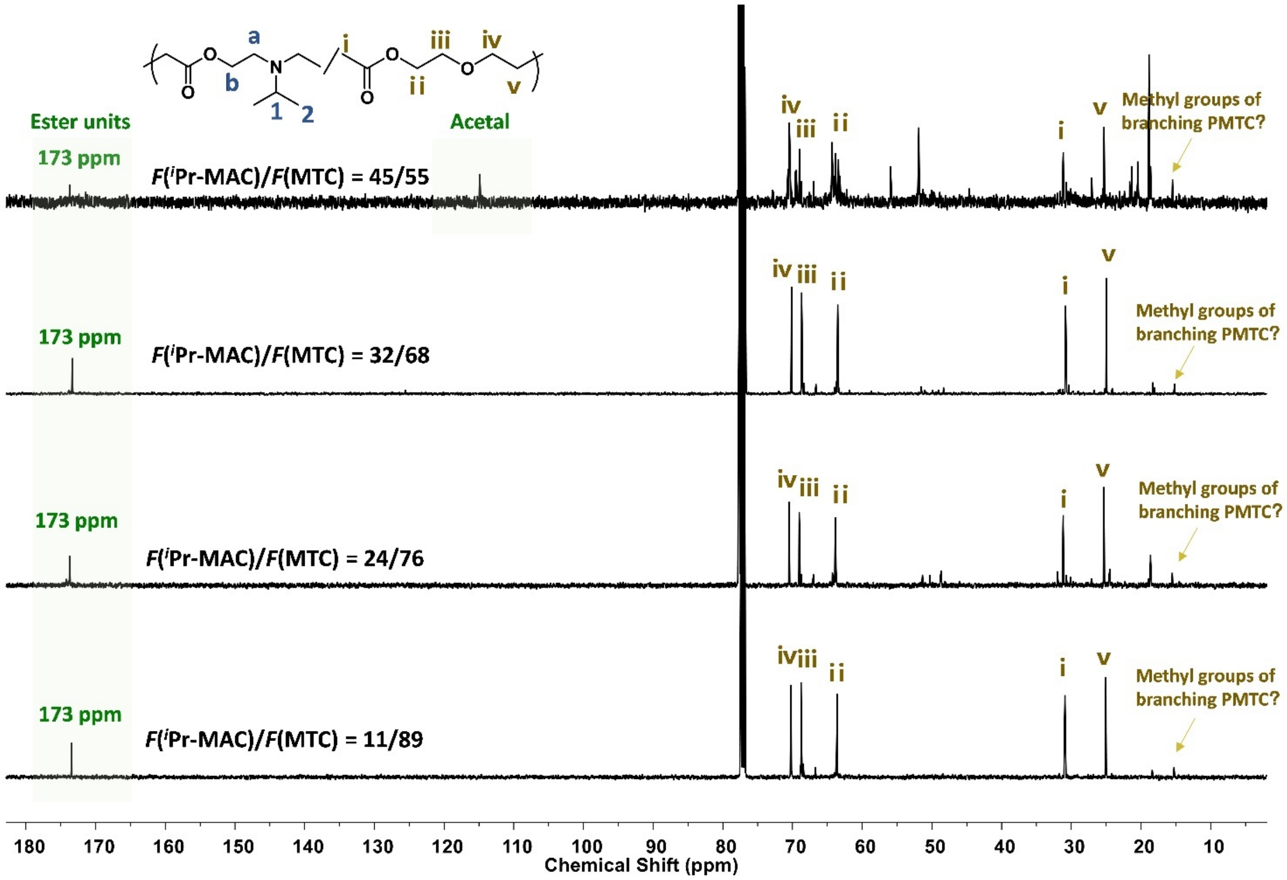

Figure S6. ^{13}C NMR spectra of purified exemplary polyester copolymers with distinct *F*(^{i}Pr-MAC)/*F*(MTC)s ranging from 11/89 to 45/55 (mol/mol). Resonances centered at 173 ppm are ascribed to the carbon atoms on the ester units of ^{i}Pr-MAC/MTC motifs. Resonances at around 114 ppm are attributed to the acetal units of ^{i}Pr-MAC.

8. 2D NMR characterizations on nanoparticles from ^{i}Pr-MAC30

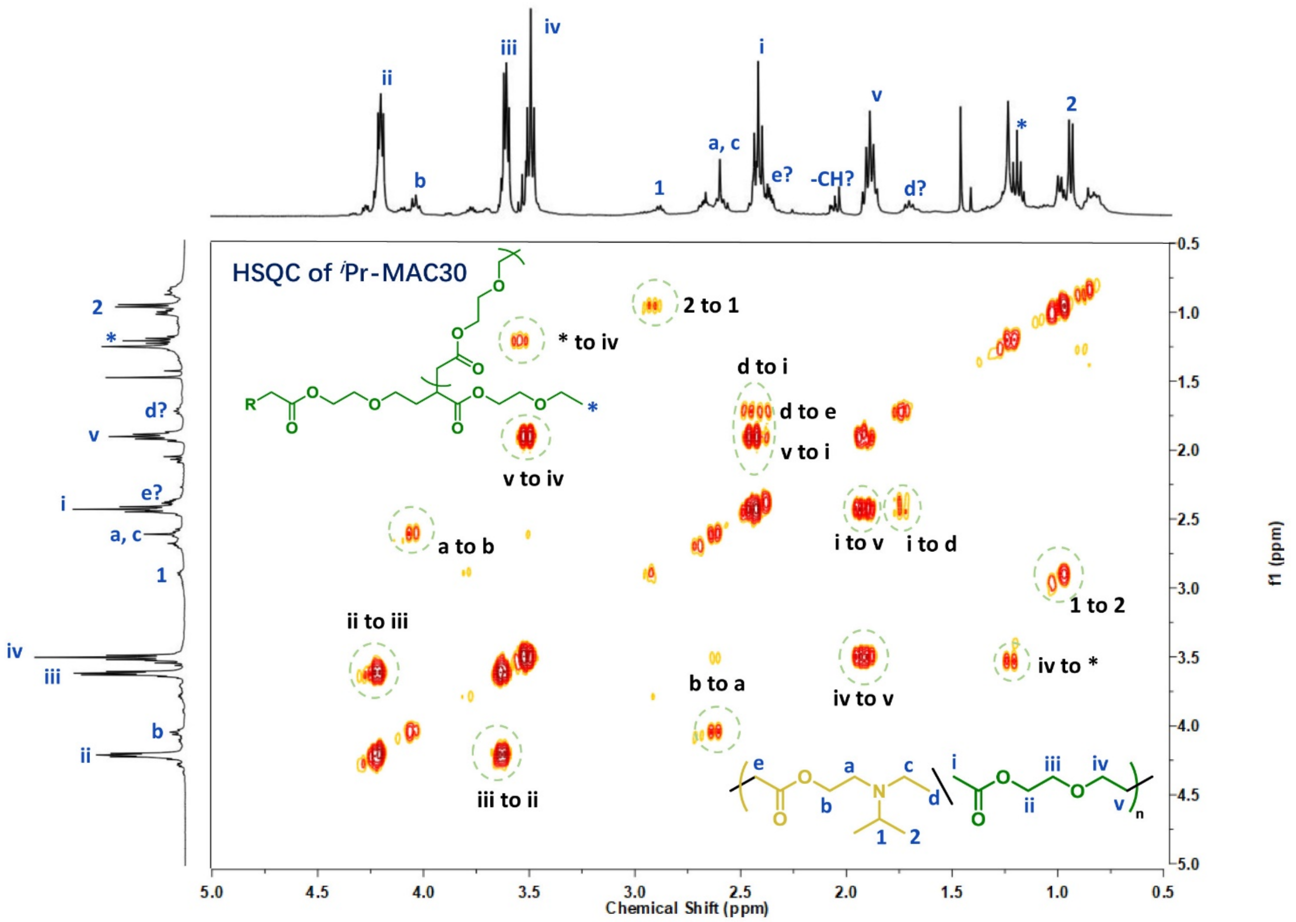

Figure S7. HSQC spectrum of polyester copolymer with a F(^{i}Pr-MAC)/F(MTC) of 31/69 (400 MHz, $CDCl_3$), affirming the proton-proton correlations within ^{i}Pr-MAC motifs, within MTC motifs, also the correlations of protons between ^{i}Pr-MAC and MTC motifs.

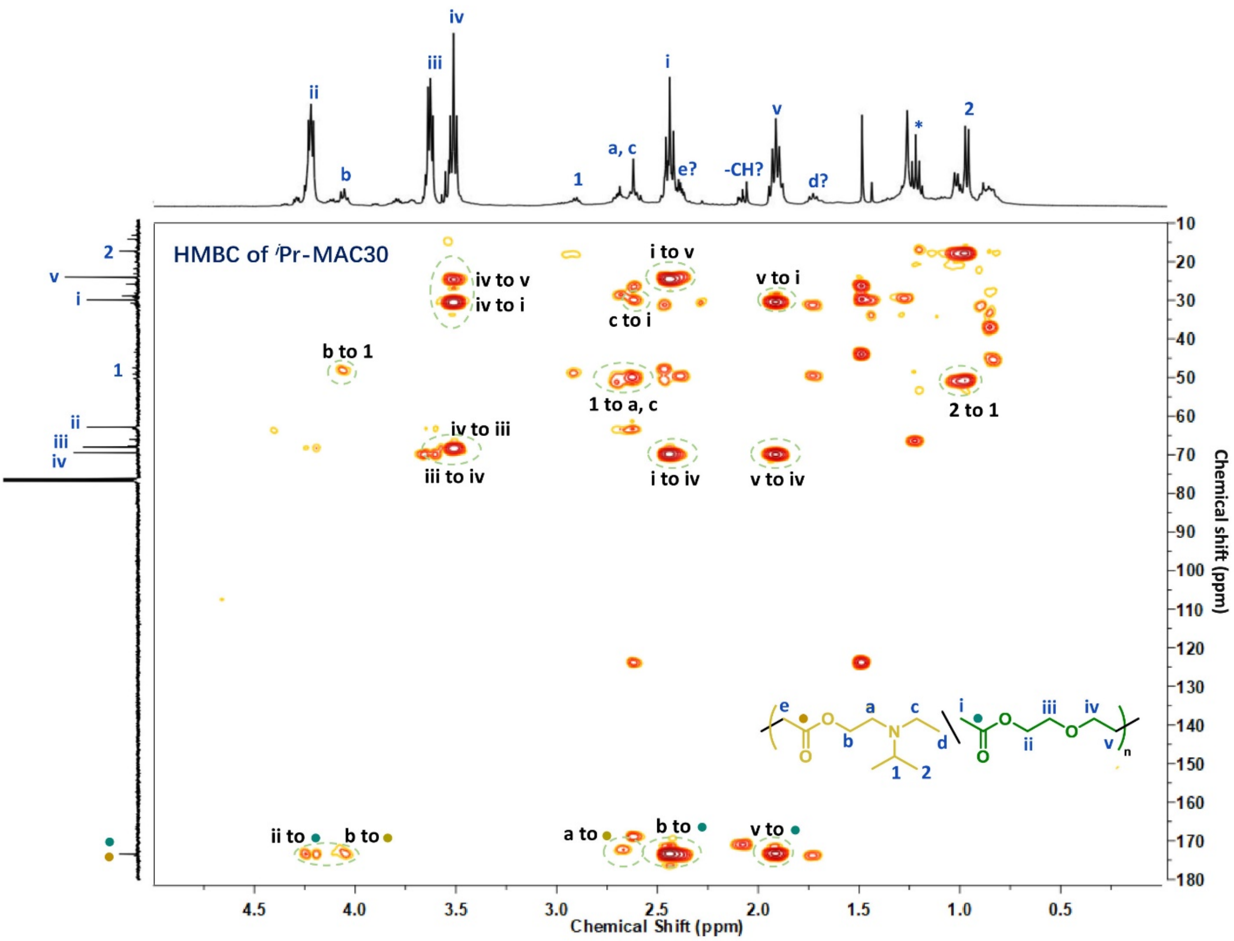

Figure S8. HMBC spectrum of polyester copolymer with a *F*(^{i}Pr-MAC)/*F*(MTC) of 31/69 (400 MHz, $CDCl_3$), indicating the carbon-proton coupling get separated by 2-4 bonds, which confirmed the existence of ring-opened structure of ^{i}Pr-MAC and MTC motifs.

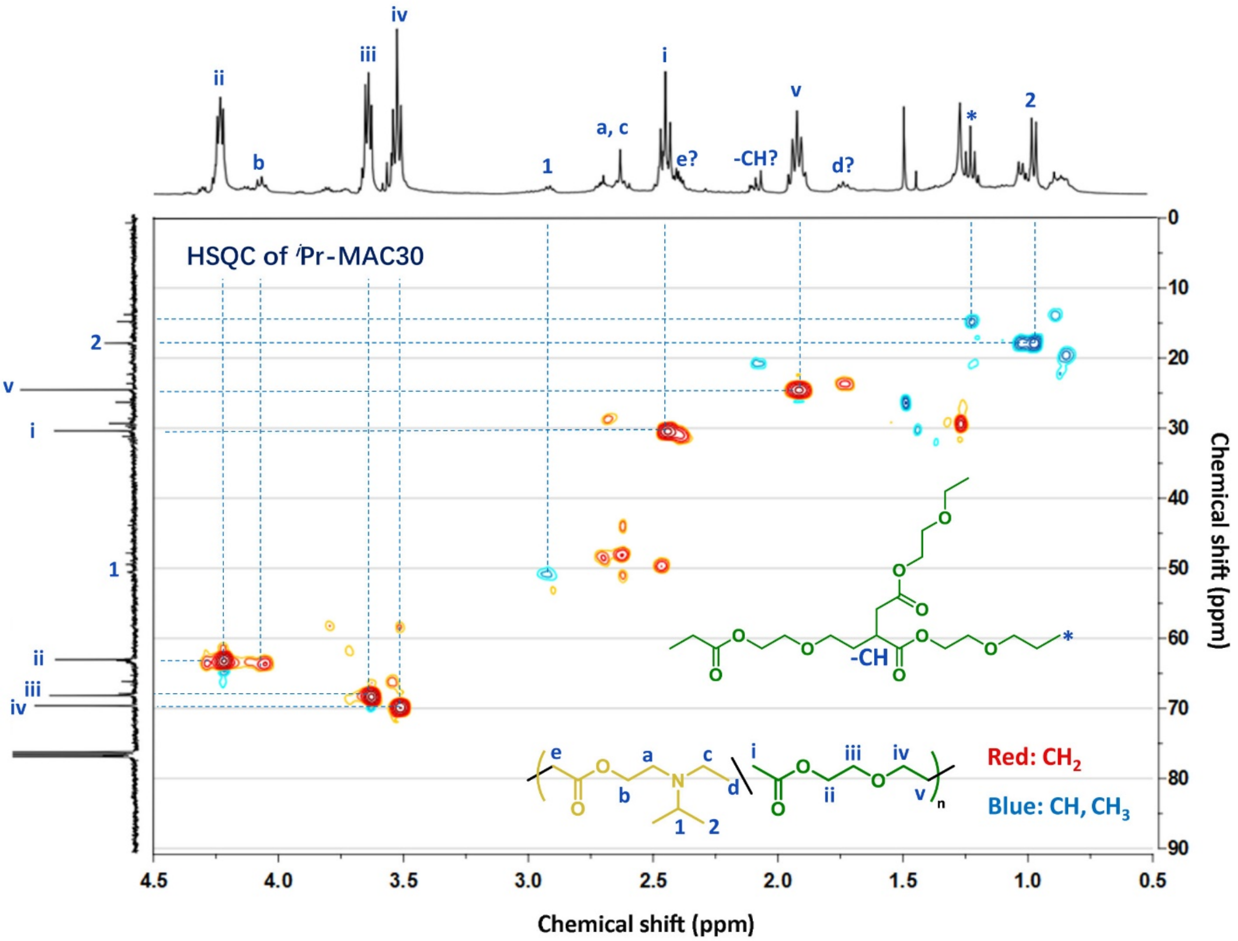

Figure S9. HSQC spectrum of polyester copolymer with a $F(^i\mathrm{Pr\text{-}MAC})/F(\mathrm{MTC})$ of 31/69 (400 MHz, $CDCl_3$), indicating the proton-carbon single bond correlations.

9. SEC characterizations on ^{i}Pr-MAC10/20/30/40

To obtain molar mass of distinct polyester copolymers, SEC measurements were performed in the presence of DMAc + LiCl as the eluent. Note that owing to the strong interactions between samples with eluent/column, the obtained M_n/M_w/PDI shall be taken into consideration with cautious.

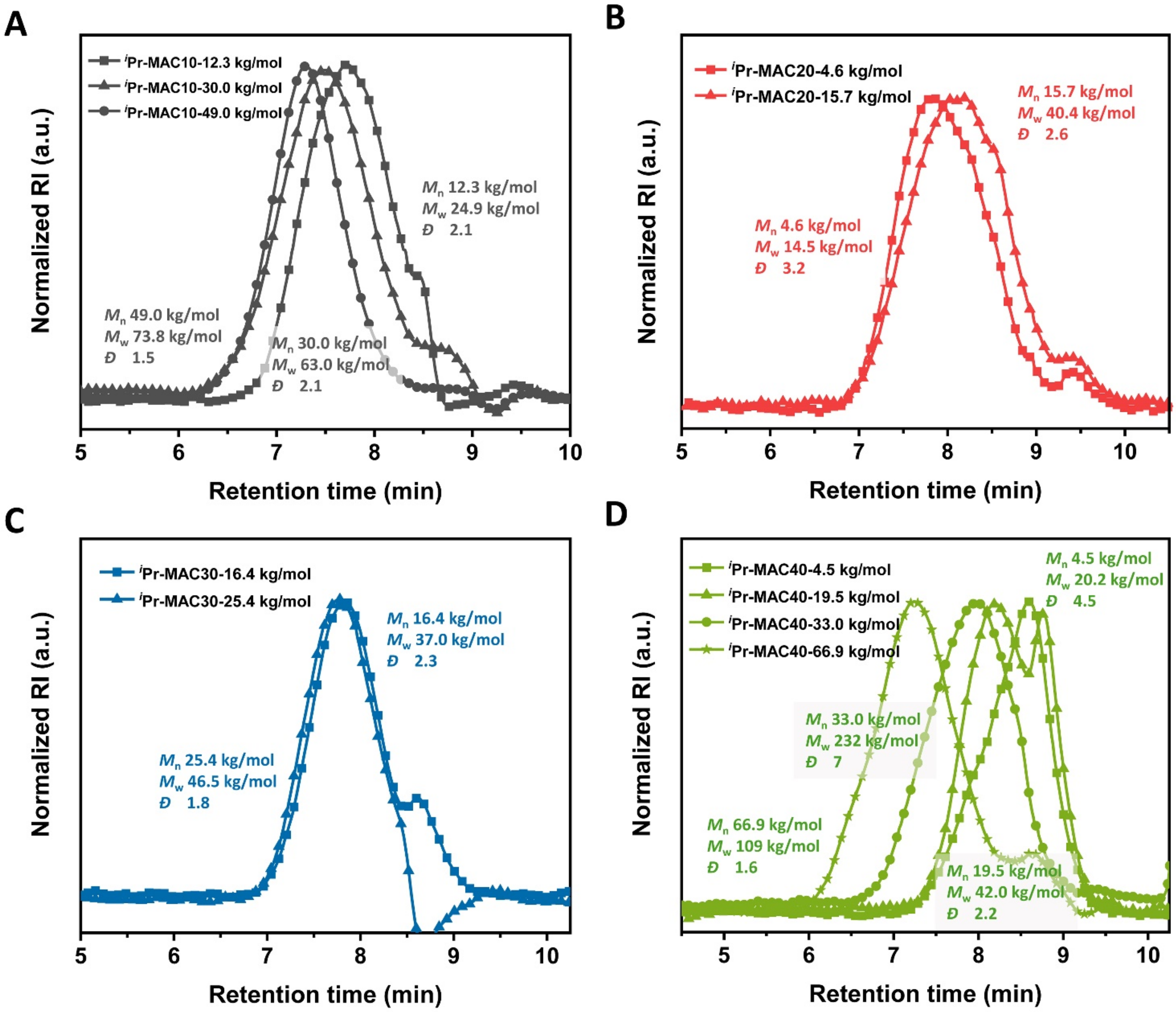

Figure S10. Normalized refractive index chromatograms of polyester copolymers iPr-MAC10/20/30/40 with distinct molar mass, characterized by SEC, with DMAc + LiCl as an eluent.

10. DOSY characterization on ^{i}Pr-MAC30

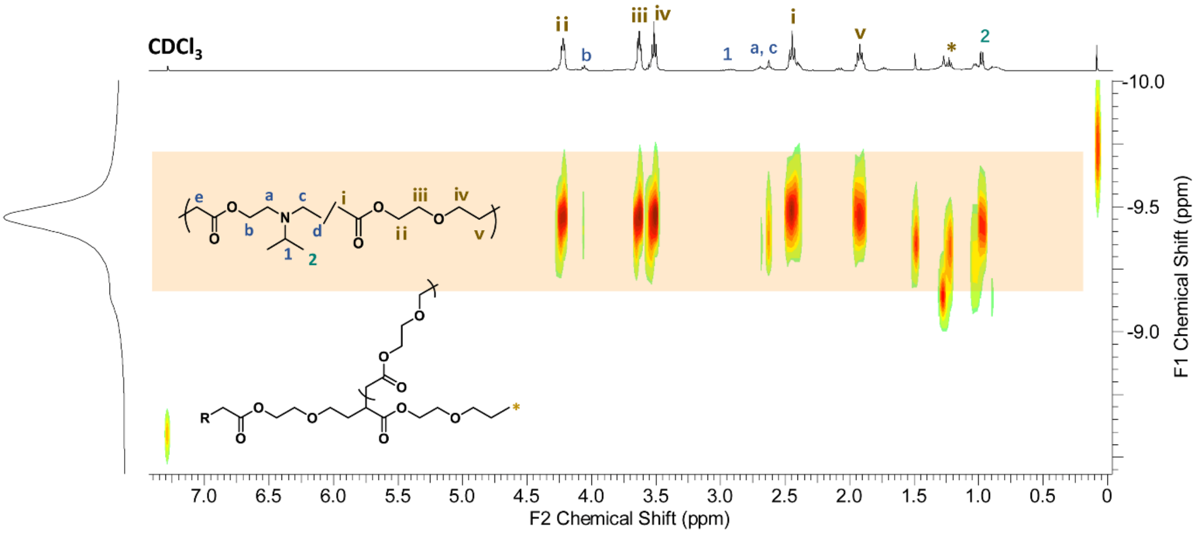

Figure S11. ^{1}H-NMR and DOSY spectra of polyester copolymer with a *F*(^{i}Pr-MAC)/*F*(MTC) of 31/69 (400 MHz, $CDCl_3$). The ^{i}Pr-MAC units (signals **1-2** and **a-c**) diffused in the similar range of MTC units (signals **i-v**), implying the successful formation of copolymers.

11. DLS characterizations on nanoparticles

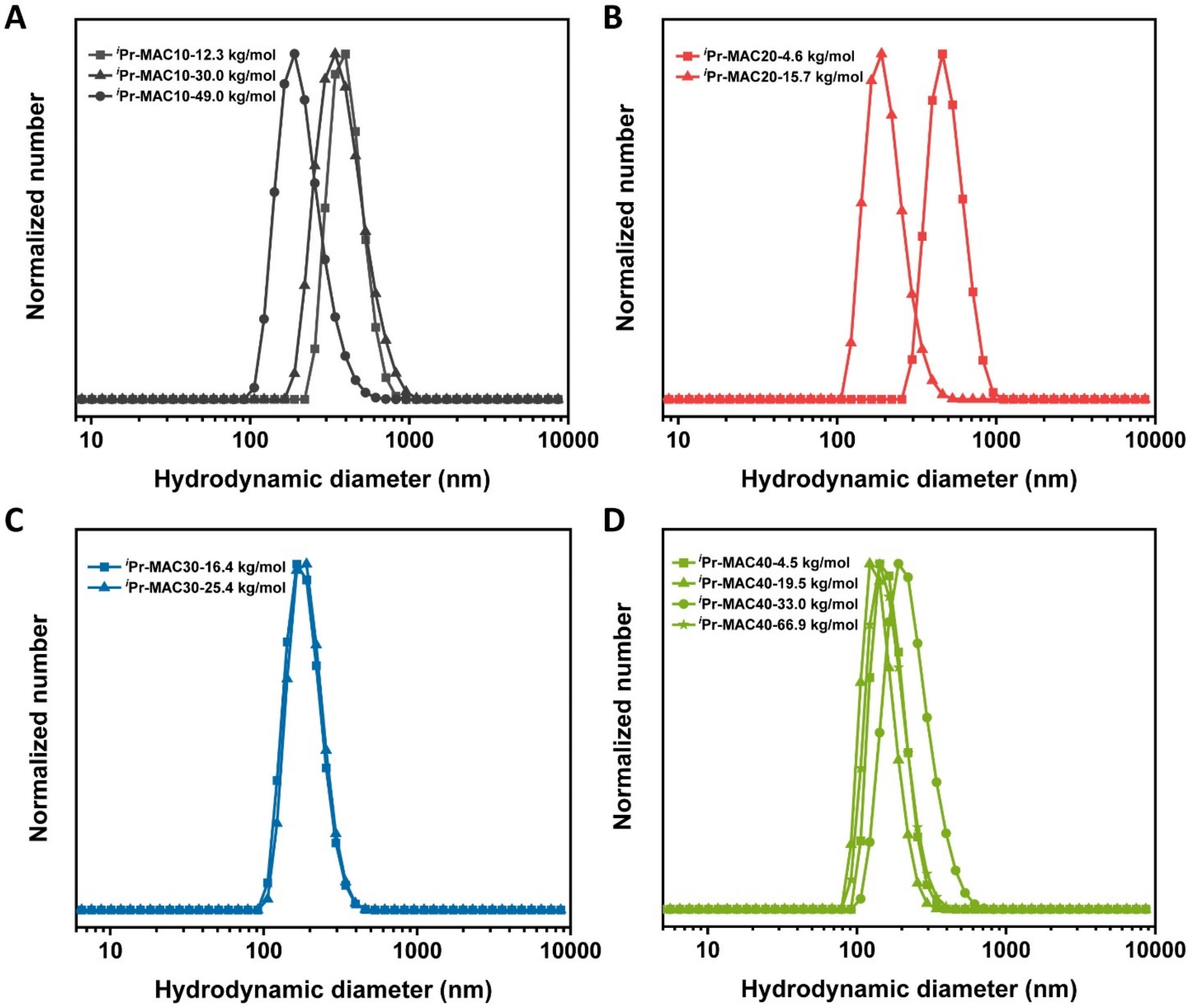

Figure S12. Number-size distributions of nanoparticles from (A-D) ^{i}Pr-MAC10/20/30/40 with distinct molar mass, characterized by DLS.

12. Colloidal suspension stability characterized by DLS and SEC

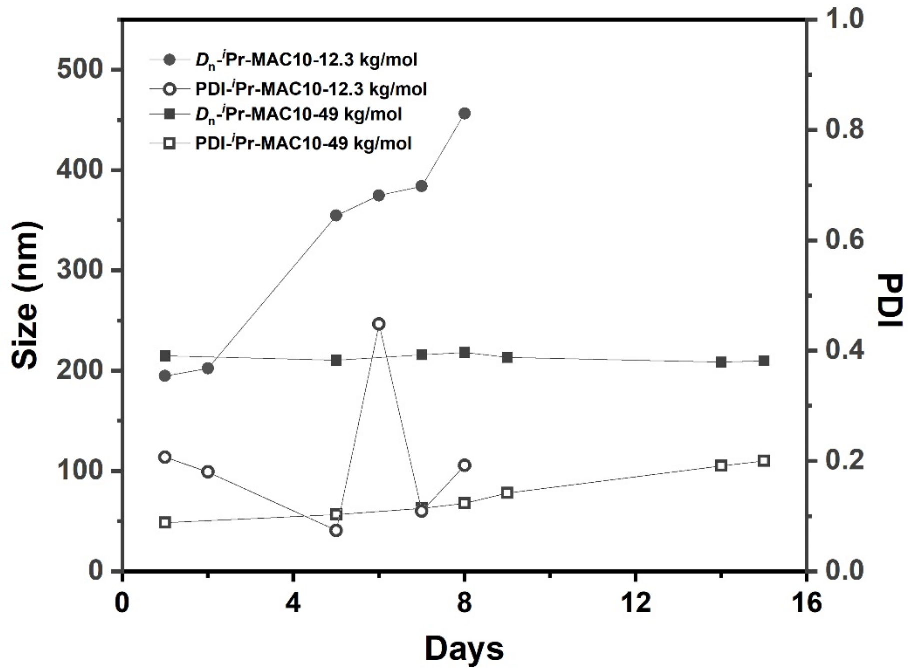

Figure S13. Changes in number-average size (D_n) and size distribution (PDI) of nanoparticles from ^{i}Pr-MAC10 (M_n of 12.3 and 49 kg/mol) during storage in water for different durations, characterized by DLS.

SEC:

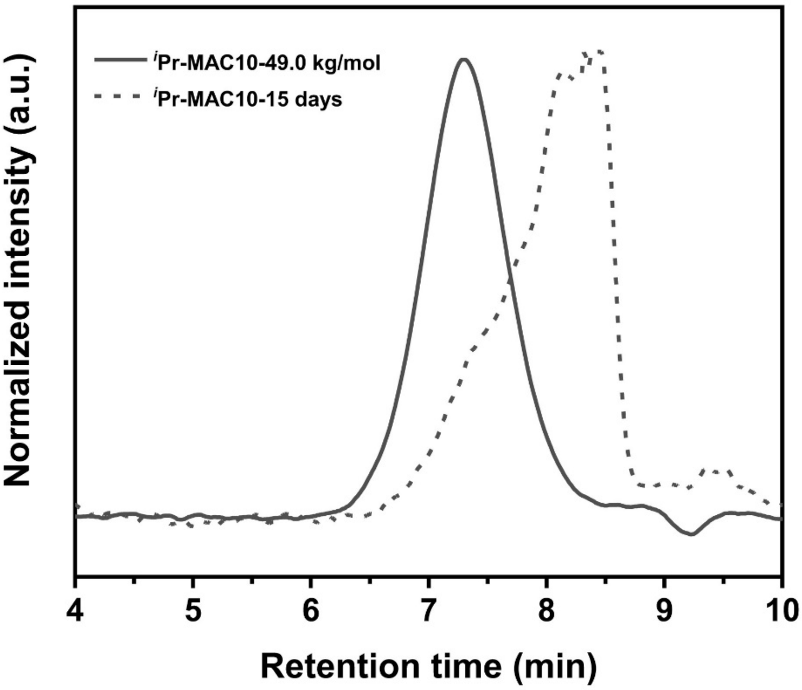

Figure S14. Normalized RI chromatograms of ^{i}Pr-MAC10 (M_n of 49 kg/mol) before (solid) and storing in water for 15 days (short dashed), characterized by SEC.

13. pH-Responsiveness of nanoparticles characterized by DLS measurements

The as-prepared nanoparticles from ^{i}Pr-MAC10/20/30/40 were subjected to pH-titration. Specifically, the pH of the nanoparticle suspension was first tuned around 8 by adding 0.01 M NaOH, and then slowly lowered to a weak acidic condition (around 5.5-6) by adding 0.01 M HCl solution. The pH of the nanoparticle suspension was measured through a pH meter. During the pH-titration process, DLS measurements were performed on the nanoparticles in different pH environments to monitor changes in size and PDI values.

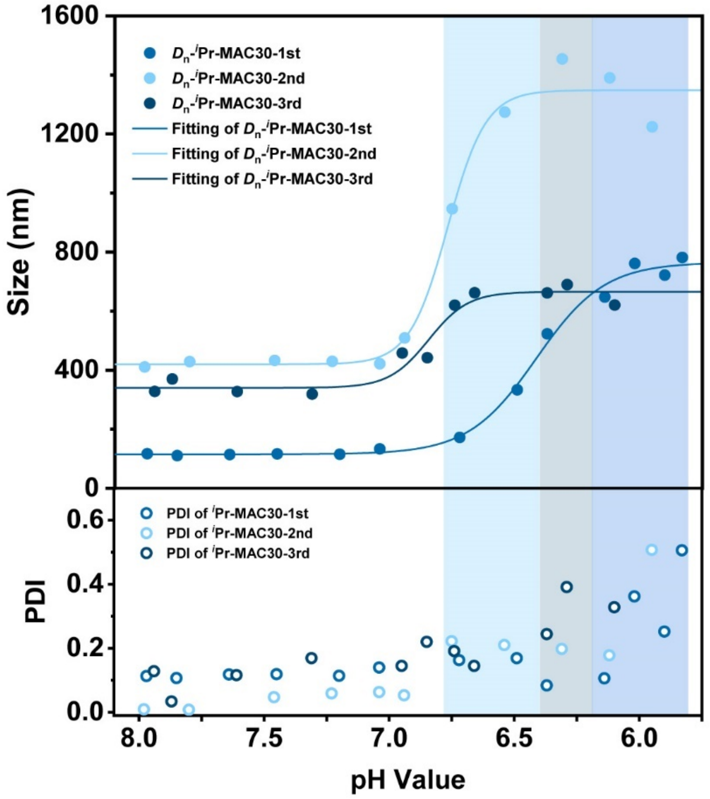

Figure S15. Evolution of D_n (solid dots) and PDI values (hollow dot) of nanoparticles from ^{i}Pr-MAC30 (M_n: 25.4 kg/mol) obtained by DLS measurements as a function of pH value. Measurements were performed in triplicate. Half-release points were recorded ranging from 6.5 to 6.9. Note that the coloured regions represent the pH ranges (from the pH value where the corresponding coloured region starts to the pH value where characterization ended) where ill-shaped correlation functions were obtained by DLS measurements, indicating the unreliable size data. To be specific, the ill-shaped correlation functions of ^{i}Pr-MAC30-1st/2nd/3rd trials were recorded when the environmental pH values were lower than 6.14, 6.74, and 6.37, respectively.

To exclude the possible swelling behaviour caused by water, control group, where deionized water was continuously added to the nanoparticle suspension without changing environmental pH value, was also subjected to DLS measurements. The size variation against environmental pH values were illustrated below with exemplary samples ^{i}Pr-MAC10 and ^{i}Pr-MAC40.

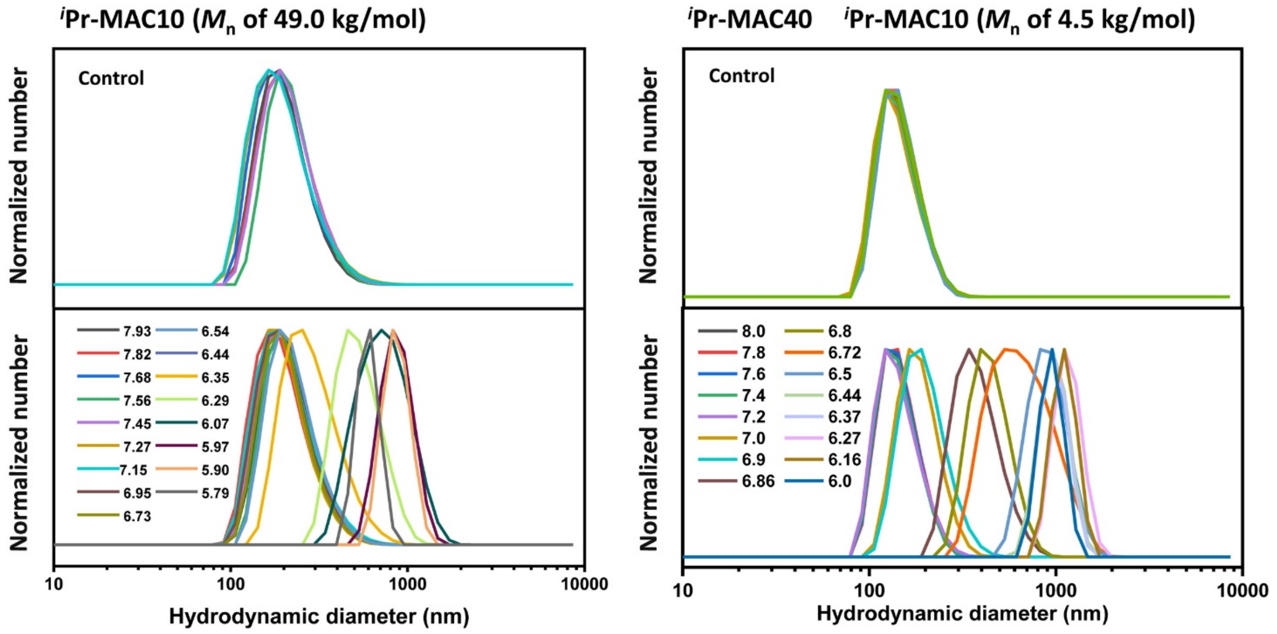

Figure S16. Size variations against pH value, characterized on nanoparticles from ^{i}Pr-MAC10 (M_n: 49 kg/mol) and ^{i}Pr-MAC40 (M_n: 4.5 kg/mol) at constant pH value (pH: 7.4, upper) and varying pH values (pH ranging around 8 to 6, lower) via DLS.

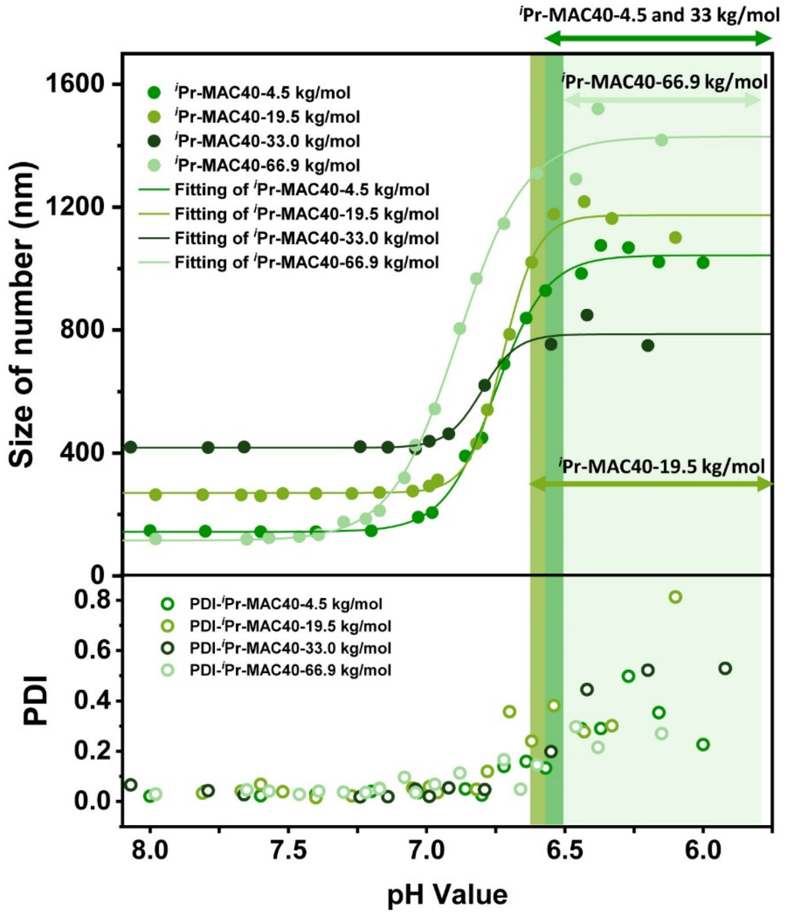

Figure S17. Evolution of D_n (solid dots) and PDI values (hollow dot) of nanoparticles from ^{i}Pr-MAC40 (M_n: 4.5, 19.5, 33.0, and 66.9 kg/mol) obtained by DLS measurements as a function of pH value. Note that the coloured regions represent the pH ranges (from the pH value where the corresponding coloured region starts to the pH value where characterization ended) where ill-shaped correlation functions were obtained by DLS measurements, indicating the unreliable size data. To be specific, the ill-shaped correlation functions of ^{i}Pr-MAC40-4.5/19.5/33.0/66.9 kg/mol trials were recorded when the environmental pH values were lower than 6.57, 6.62, 6.57 and 6.46, respectively.

pH-Responsiveness of Nile red-loaded nanoparticles characterized by DLS measurements

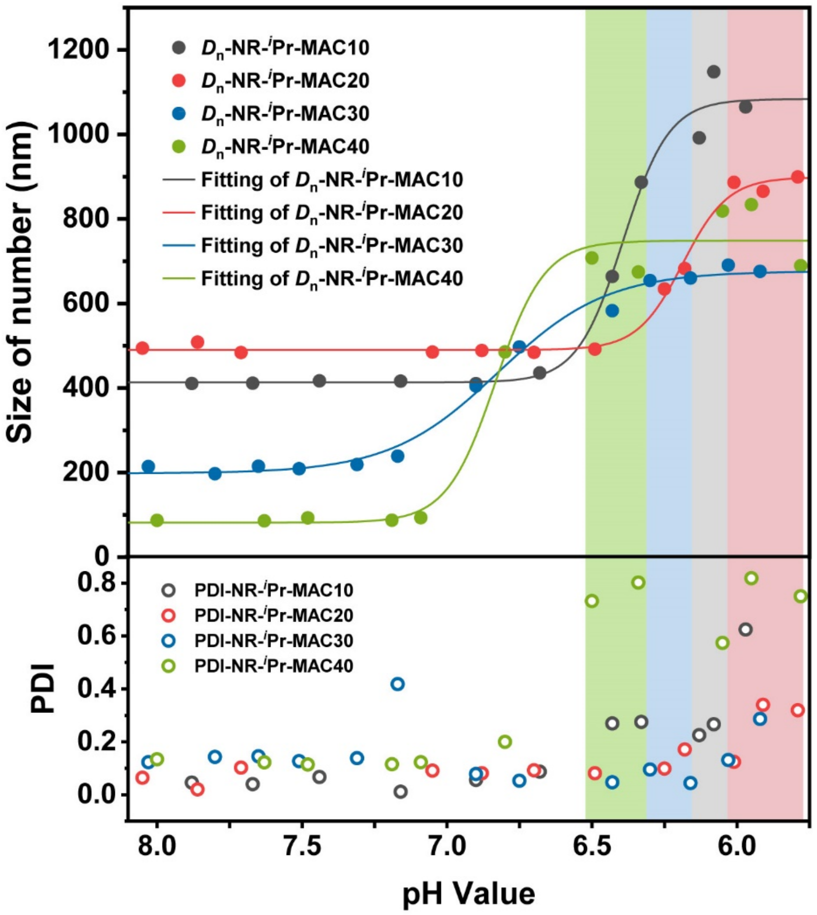

Figure S18. Evolution of D_n (solid dots) and PDI values (hollow dot) of Nile red-loaded nanoparticles from ^{i}Pr-MAC10/20/30/40 obtained by DLS measurements as a function of pH value. Note that the coloured regions represent the pH ranges (from the pH value where the corresponding coloured region starts to the pH value where characterization ended) where ill-shaped correlation functions were obtained by DLS measurements, indicating the unreliable size data. To be specific, the ill-shaped correlation functions of ^{i}Pr-MAC10/20/30/40 were recorded when the environmental pH values were lower than 6.13, 6.01, 6.30 and 6.50, respectively.

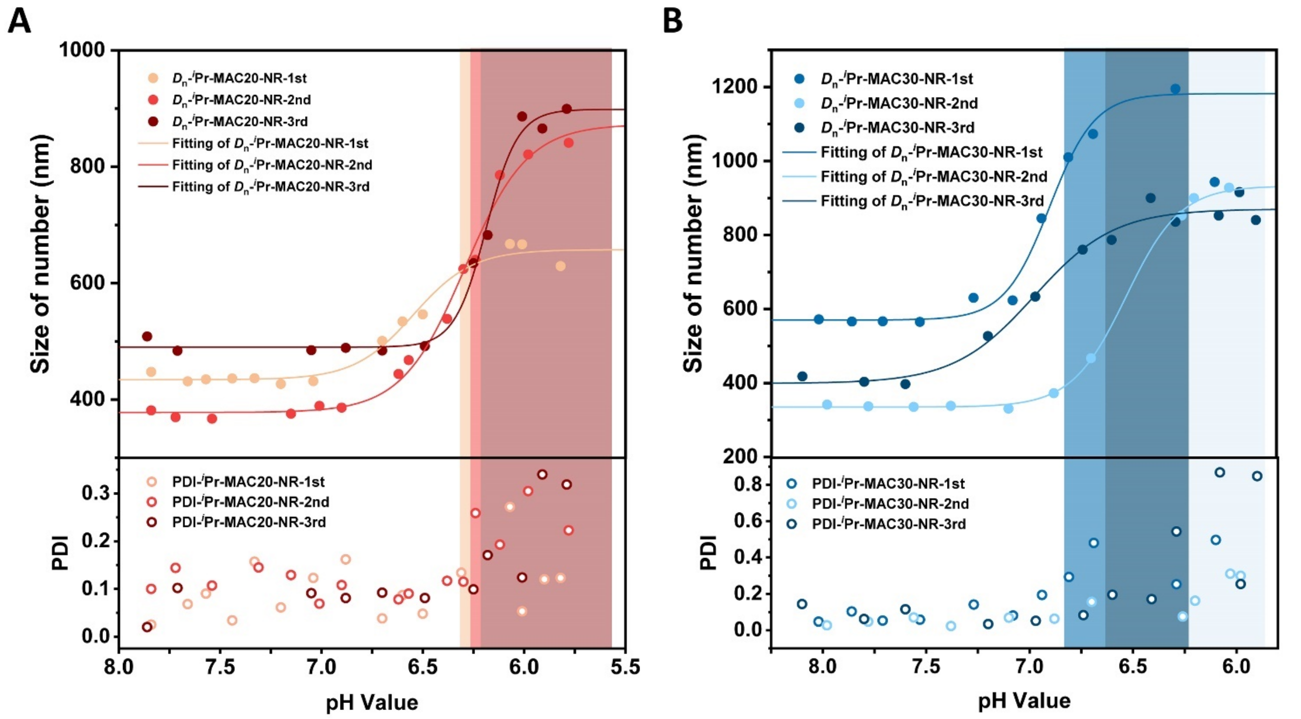

Figure S19. Evolution of D_n (solid dots) and PDI values (hollow dot) of Nile red-loaded nanoparticles from (A) ^{i}Pr-MAC20 (M_n: 4.5 kg/mol) and (B) ^{i}Pr-MAC30 (M_n: 25.4 kg/mol) obtained by DLS measurements as a function of pH value. Measurements were performed in triplicate. Half-swelling points were recorded ranging from 6.2 to 6.5 and 6.5 to 6.9 for Nile red-loaded nanoparticles from ^{i}Pr-MAC20 and 30, respectively. Note that the coloured regions represent the pH ranges (from the pH value where the corresponding coloured region starts to the pH value where characterization ended) where ill-shaped correlation functions were obtained by DLS measurements, indicating the unreliable size data. To be specific, the ill-shaped correlation functions of ^{i}Pr-MAC20-NR-1st/2nd/3rd trials were recorded when the environmental pH values were lower than 6.31, 6.24, and 6.18, respectively. For ^{i}Pr-MAC30-NR-1st/2nd/3rd trials, 6.81, 6.20, and 6.60, respectively.

14. pH-Triggered Nile red release characterized by fluorescence spectroscopy

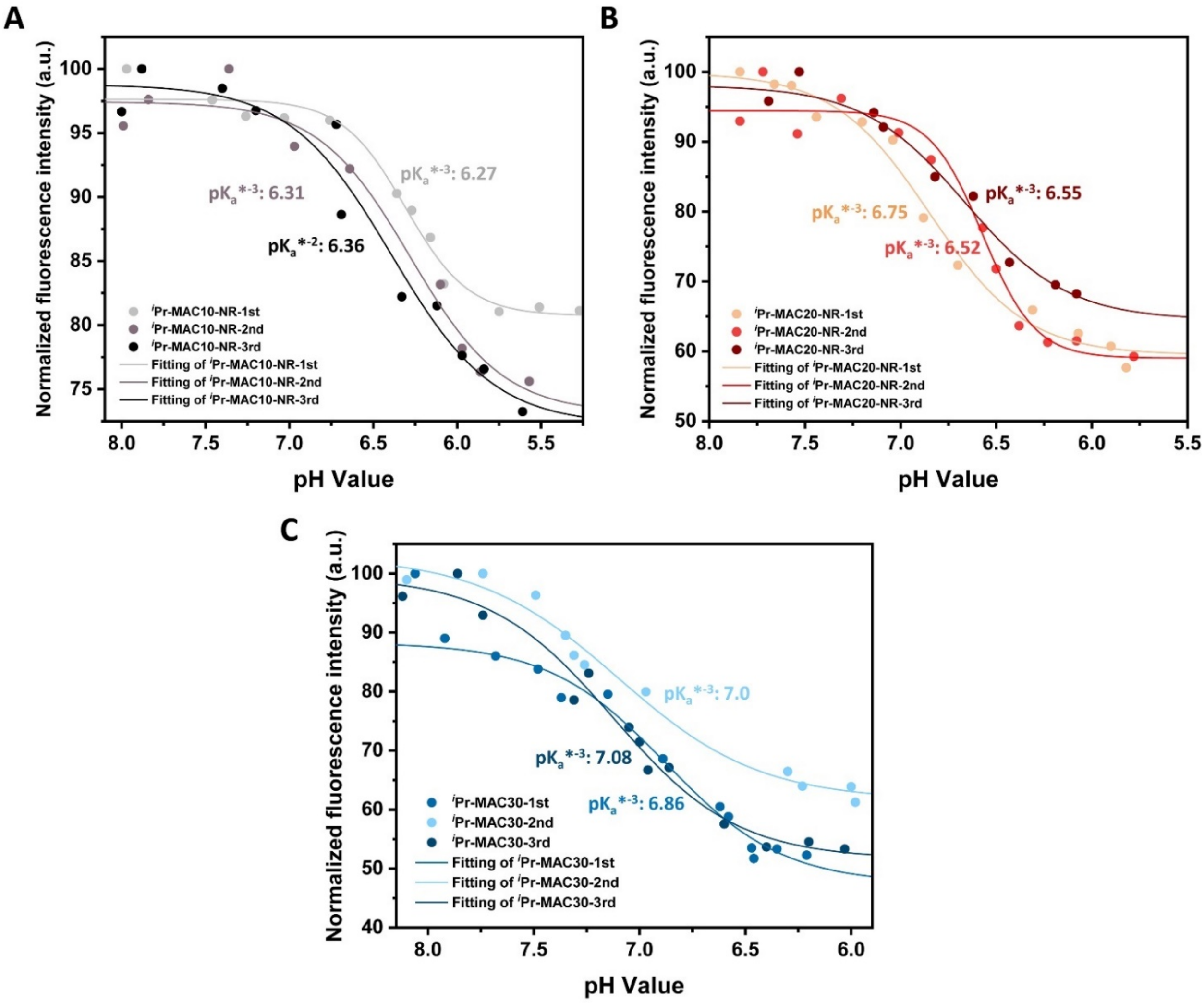

Figure S20. Fluorescence intensity decline pattern of Nile-red loaded nanoparticles from (A) ^{i}Pr-MAC10 (M_n of 12.3 kg/mol), (B) ^{i}Pr-MAC20 (M_n of 4.6 kg/mol), and (C) ^{i}Pr-MAC30 (M_n of 25.4 kg/mol) against environmental pH values, and the corresponding half-release points. Measurements were performed in triplicate.

Table S2. Average transition points and deviations of NPs prepared from ^{i}Pr-MAC10/20/30/40 with distinct molar mass, characterized by different means.

	pK_a^*-D1[a]	pK_a^*-D2[b]	pK_a^*-F[d]
^{i}Pr-MAC10	6.3±0.2	6.4±0.0[c]	6.4±0.1
^{i}Pr-MAC20	6.5±0.3	6.5±0.3	6.6±0.1
^{i}Pr-MAC30	6.7±0.2	6.8±0.2	7.0±0.1
^{i}Pr-MAC40	6.9±0.2	6.8±0.1	7.0±0.1

[a] pK_a^*-D1, half-swelling point, defined as the pH value at which the size reaches half of its increase, characterized by DLS; [b] pK_a^*-D2, half-swelling point of Nile red-loaded NPs, defined as the pH value at which the size reaches half of its increase, characterized by DLS; [c] pK_a^*-F, half-release point, defined as the pH points where the fluorescence intensity reaches 50 % of the total reduction, characterized by fluorescence spectroscopy.

15. Enzymatic degradation characterized by Tecan plate reader

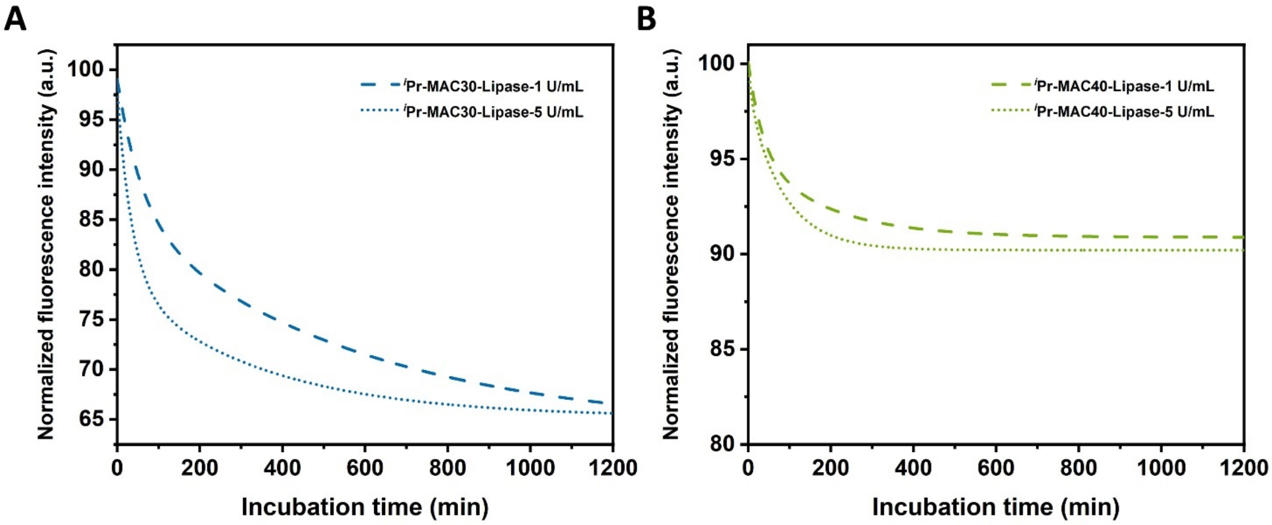

Figure S21. Fluorescence intensity decline pattern of Nile-red loaded nanoparticles from (A) ^{i}Pr-MAC30 (M_n of 25.4 kg/mol), and (B) ^{i}Pr-MAC40 (M_n of 33.0 kg/mol) against incubation time. Nile red-loaded nanoparticle suspension was treated with Lipase from *Pseudomonas cepacian* (1 U/mL and 5 U/mL).

16. Cellular uptake and gene expression

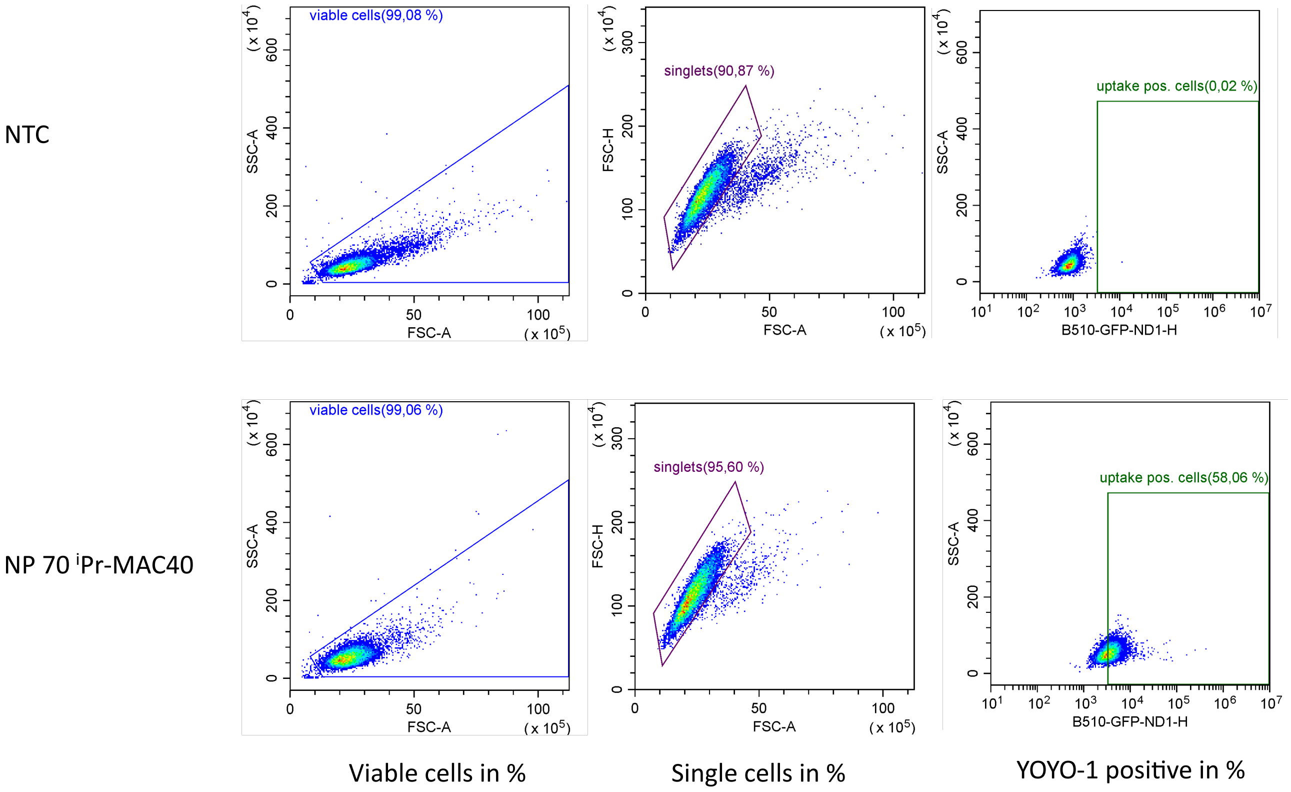

Figure S22 Gating strategy (left: viable cells, middle: single cells, right: YOYO-1 positive cells). Upper row for no template control (NTC) and lower row exemplary for the NP70 *i*Pr-MAC40 formulation.

References

[1] J. Stumpff, G. von Dassow, M. Wagenbach, C. Asbury, L. Wordeman, *Developmental Cell* **2008**, 14, 252-262.
